# Revolution of AAV in Drug Discovery: From Delivery System to Clinical Application

**DOI:** 10.1002/jmv.70447

**Published:** 2025-06-19

**Authors:** Ling Yin, Hongliang He, Hongliang Zhang, Yuhua Shang, Chengbo Fu, Songquan Wu, Tengchuan Jin

**Affiliations:** ^1^ Center of Disease Immunity and Intervention, College of Medicine Lishui University Lishui China; ^2^ College of Medicine, University of Florida Gainesville Florida USA; ^3^ Division of Infectious Diseases and Geographic Medicine, Department of Internal Medicine University of Texas Southwestern Medical Center Dallas Texas USA; ^4^ Institute of Health and Medicine Hefei Comprehensive National Science Center Hefei Anhui China; ^5^ Biomedical Sciences and Health Laboratory of Anhui Province University of Science & Technology of China Hefei Anhui China; ^6^ Laboratory of Structural Immunology, National Key Laboratory of Immune Response and Immunotherapy, Division of Life Sciences and Medicine University of Science and Technology of China Hefei Anhui China; ^7^ Anhui Genebiol Biotech. Ltd. Hefei Anhui China; ^8^ Clinical Research Hospital of Chinese Academy of Sciences (Hefei) University of Science and Technology of China Hefei Anhui China; ^9^ Key Laboratory of Anhui Province for Emerging and Reemerging Infectious Diseases Hefei Anhui China; ^10^ Division of Life Sciences and Medicine, Department of Obstetrics and Gynecology, The First Affiliated Hospital of USTC, Center for Advanced Interdisciplinary Science and Biomedicine of IHM University of Science and Technology of China Hefei Anhui China

**Keywords:** AAV, clinical application, delivery system, drug discovery

## Abstract

Adeno‐associated virus (AAV) is a non‐enveloped DNA virus infecting a wide variety of species, tissues, and cell types, which is recognized as a safe and effective method for delivering therapeutic transgenes. AAV vector is the most popular viral gene delivery system in clinical delivery systems with unique and multiple advantages, such as tissue tropism, transduction specificity, long‐lasting gene expression, low immune responses, and without host chromosome incorporation. Till now, four AAV‐based gene therapy drugs have already been approved by the US Food and Drug Administration (FDA) or European Medicines Agency (EMA). Despite the success of AAV vectors, there are still some remaining challenges that limit further usage, such as poor packaging capacity, low organ specificity, pre‐existing humoral immunity, and vector dose‐dependent toxicity. In the present review, we address the different approaches to optimize AAV vector delivery system with a focus on capsid engineering, packaging capacity, and immune response at the clinical level. The review further investigates the potential of manipulating AAV vectors in preclinical applications and clinical translation, which emphasizes the challenges and prospects in viral vector selection, drug delivery strategies, immune reactions in cancer, neurodegenerative disease, retinal disease, SARS‐CoV‐2, and monkeypox. Finally, it forecasts future directions and potential challenges of artificial intelligence (AI), vaccines, and nanobodies, which emphasizes the need for ethical and secure approaches in AAV application.

## 1 Introduction

Adeno‐associated virus (AAV) belongs to the Dependoparvovirus genus of Parvovirinae subfamily within Parvoviridae family, which was first reported as a contaminant of adenovirus in 1965 [1]. AAV is a non‐enveloped single‐stranded DNA parvovirus with a 4.7 kb genome comprising two open reading frames (ORFs): rep, which encodes four non‐structural replicative proteins (Rep78, Rep68, Rep52, and Rep40) involved in viral replication, packaging, and genomic integration, and cap, which encodes three viral capsid proteins (VP1, VP2, and VP3) involved in viral capsid formation and viral gene delivery [2]. AAV genome is flanked by two 145 bp inverted terminal repeats (ITRs), with a rep‐protein binding site (RBS) and a terminal resolution site (TRS), form T‐like hairpins with 125 bp and form D‐sequences with 20 bp [3]. To date, over 10 serotypes and 100 variants of AAV have been characterized for diverse tropism characteristics based on various cellular primary and co‐receptors, with serotypes 1, 3, 4, 7, 8, 10, and 11 isolating from non‐human primates (NHPs), and serotypes 2, 5, 6, and 9 isolating from humans [4, 5].

AAV has become the leading gene therapy for treating a variety of diseases, such as hepatocellular carcinoma (HCC), X‐linked cone dystrophy, hemoglobinopathies, and Alzheimer's disease (AD) [6, 7, 8, 9]. Till now, four AAV therapies, Luxturna, Zolgensma, Hemgenix, and Elevidys, have been, respectively, approved for the treatment of retinal dystrophy, spinal muscular atrophy (SMA), hemophilia B, and Duchenne muscular dystrophy by the Food and Drug Administration (FDA) [10]. However, there are still several barriers to hinder AAV applications, such as poor vector biodistribution, reduced target efficiency, limited transduction safety, and potential immune response [11]. These concerns motivate the modification of AAV delivery system to achieve high transduction efficiency, target potency, and selective expression [12].

Drug discovery is laborious, costly, and time‐consuming, consuming more than 12 years and 2.5 billion dollars with a 90% failure rate [13]. Drug discovery includes trial‐and‐error experimentation, drug‐material interactions, and drug delivery systems, which could be optimized by analyzing structure‐function relationships, conducting rational design, and implementing high‐throughput screens [14]. Artificial Intelligence (AI) combines novel computational techniques with conventional scientific exploration to breakthrough enduring obstacles in drug discovery, which includes target identification, drug design, pharmacological purposing, chemical synthesis, and property prediction [15]. Based on current understanding of viral biology and platform state, the review provides a detailed overview of the optimized strategies to develop AAV vector delivery system, such as capsid engineering, packaging capacity, and immune response. Due to unique biological and biophysical properties, AAV‐mediated gene therapy applications in preclinical and clinical studies of multiple diseases (cancer, neurodegenerative disease, and retinal disease) are also summarized in the review. The review also illustrates the incorporation of AAV into clinical‐stage gene therapy candidates in SARS‐CoV‐2, as well as the applications of AAV to future therapeutic opportunities in monkeypox. Finally, we also provide future AAV research trends.

## 2 AAV Delivery System

**Figure 1:**
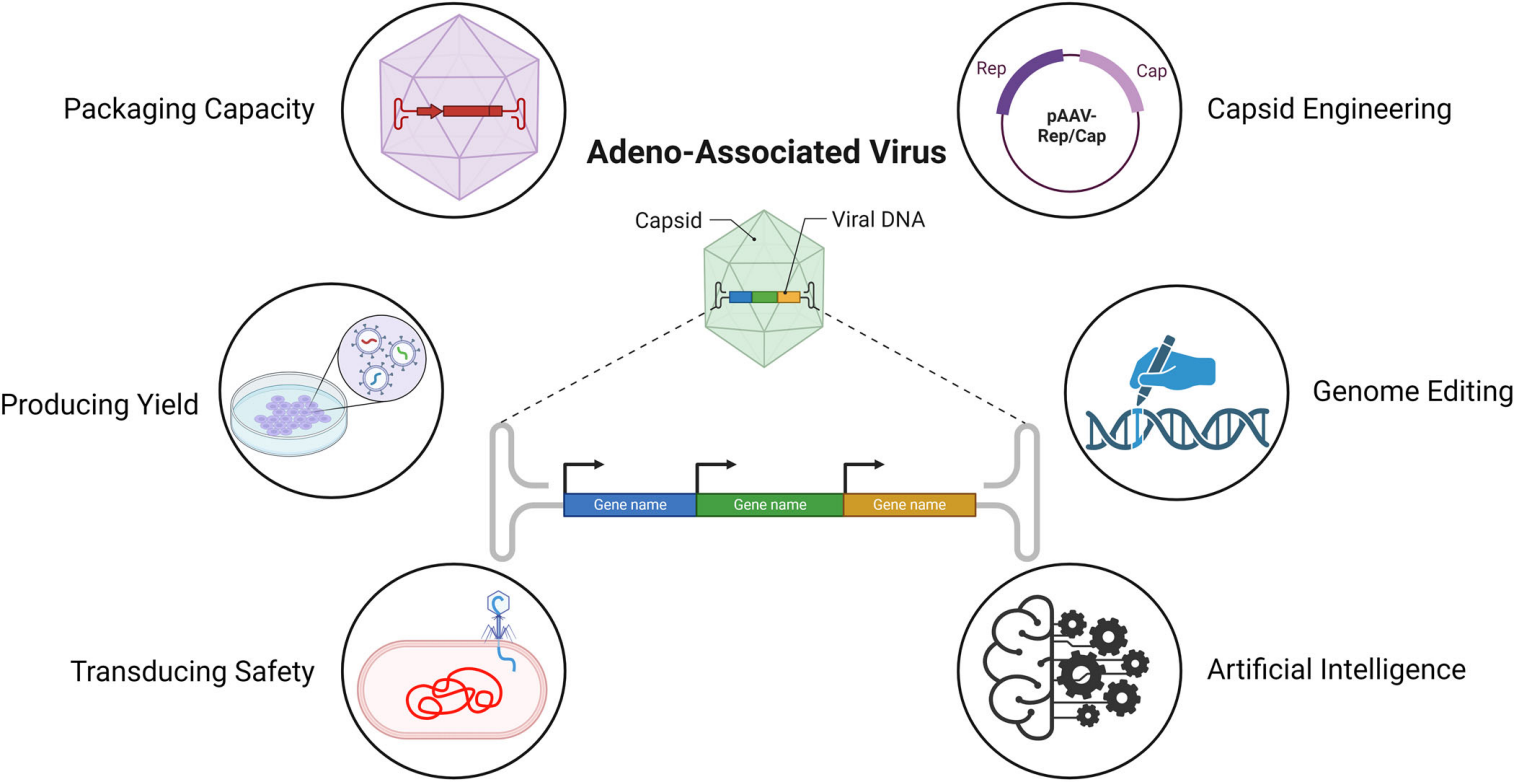
Schematic overview of adeno‐associated virus (AAV) packaging and engineering. The diagram illustrates key components and processes involved in AAV production, including packaging capacity, producing yield, and transducing safety. The figure also shows advanced techniques like capsid engineering, genome editing, and the integration of artificial intelligence in AAV research.

Recent advances in high‐throughput DNA synthesis, multiplexing, and sequencing technologies have accelerated and modified AAV delivery system [16], such as capsid properties, production yield, packaging efficiency, biodistribution potential, and transduction safety (Figure 1).

### 2.1 Capsid Engineering

**Figure 2:**
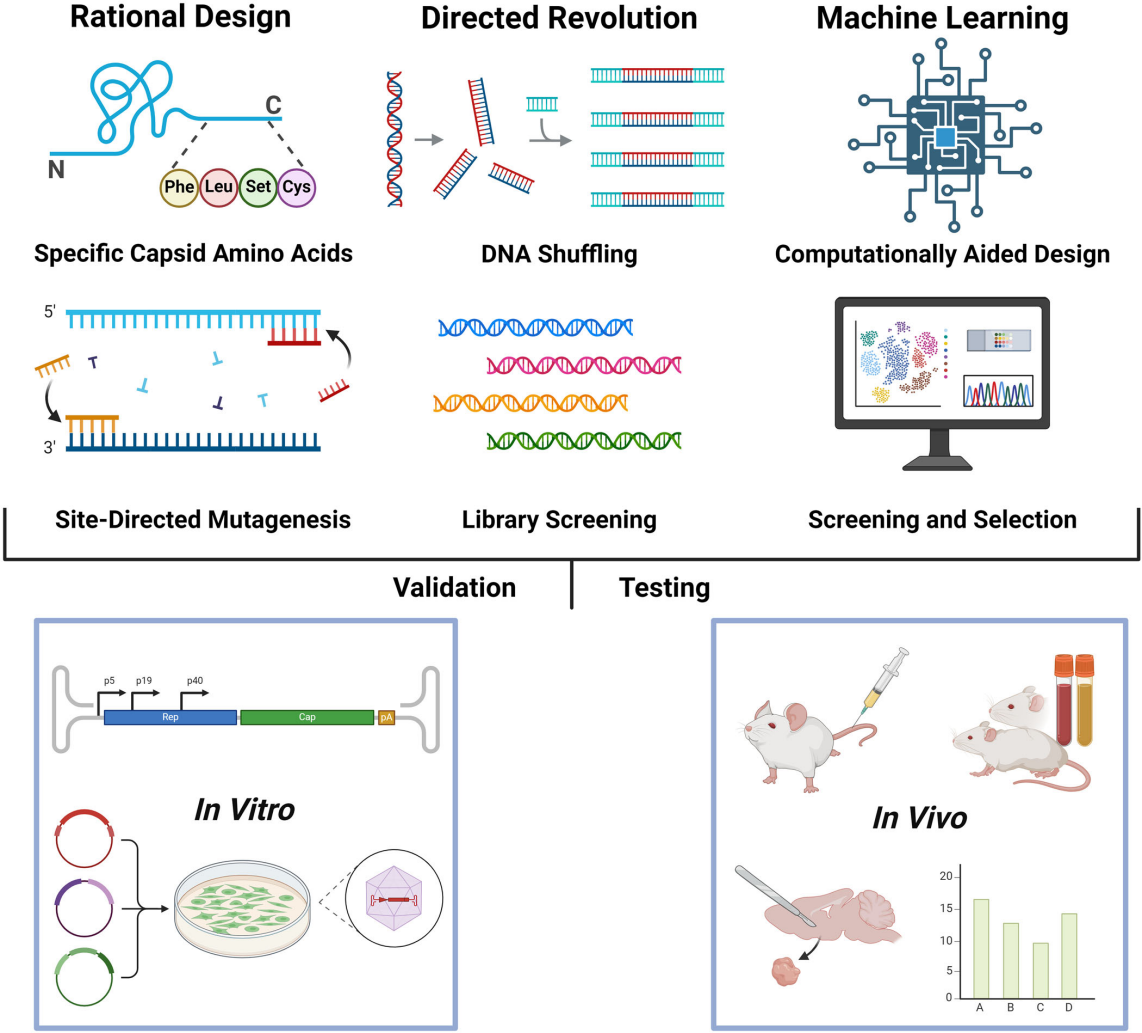
The strategies for optimizing AAV capsid engineering. AAV capsids could be engineered through various methods, including rational design, directed evolution, and machine learning. Rational Design focuses on targeted modifications based on structural or functional insights. Directed Evolution (bottom) presents a high‐throughput mutant library. Machine learning is employed to analyze variant patterns and optimize screening efficiency. These diversified AAV capsids are then validated and tested within a chosen system of interest, In Vitro and In Vivo.

Due to a lack of tissue specificity and tropism spectra, AAV capsids are still not sufficient for the successful application and development of clinically approved therapy [17]. To overcome tropism limitations, several approaches have been applied to date, such as rational design, directed evolution, and machine learning (ML) (Figure 2).

The rational design approach, the first method applied for AAV capsid modification, utilizes the direct alteration of capsid structure via peptide or protein insertions, which is based on the understanding of viral capsid and receptor interactions [18]. Rational design approaches for AAV capsid engineering include several strategies [19], such as capsid point mutations, domain swapping, and chemical modification for modulating, improving, optimizing, and enhancing the vector therapeutic efficiency, which create new generations of vectors for human gene therapy (Table 1). The clade F AAV hematopoietic stem cell 16 (AAVHSC16) residues 501, 505, and 706 show minimal liver transduction and reduced galactose binding following peripheral injection [20]. The non‐clade‐F AAVrh74 and AAV8 serotypes capsids, which are rationally designed at the positions corresponding to AAVHSC‐16 (501I, 505R, and 706C), showed significantly reduced transduction in the liver, which could be used to de‐target AAV therapies [21]. Based on angiotensin‐converting enzyme 2 (ACE2) receptor‐mediated cell entry function, soluble human ACE2 (shACE2) and 24‐83aa fragment of dog ACE2 (dACE2_24‐83_) had high affinity to the receptor binding domain (RBD) of severe acute respiratory syndrome coronavirus 2 (SARS‐CoV‐2) variants. Rational design of AAVrh10‐vectored shACE2 or dACE2_24‐83_ constructs broadly blocks the cell entry of SARS‐CoV‐2 variants by increasing binding affinity and decreasing side effects, which could serve as a promising therapeutic strategy against SARS‐CoV‐2 variants infections [22]. Among nine variable regions (VRs) of AAV virion capsid proteins, VRI plays an important role in AAV transduction, biodistribution, and Nab recognition in different tissues [23, 24]. Novel AAV6 capsids by VRI swapping through rational design exhibit high local transduction, low neutralizing antibody formation, and limited off‐target effects, which laid a foundation for potential clinical applications of optimized joint gene therapy [25].

**Table 1:** AAV capsid engineering in drug discovery and disease treatment.

Serotype	Origin	Glycan receptor	Co‐receptor	Tissue tropism	Drug discovery	Reference
AAV1	Primate	N‐linked sialic acid	Unknown	Skeletal muscle, CNS, pancreas, lung, retina,	AAV1‐BBB6 AAV1‐BBB28 AAV1‐BBB31	[26]
AAV2	Human	HSPG	FGFR1 HGFR LamR, CD9 tetraspanin	Smooth muscle, CNS, skeletal muscle, liver, kidney	AAV2tYF‐TV AAV2.MB453 AAV2tYF‐CNGA3/B3 AAV2tYF‐RPGR AAV2tYF‐hRS1 AAV2‐REP1 AAV2‐hRPE65 AAV2‐hRPE65v2‐101/301 AAV2‐ND4 scAAV2‐P1ND4v2 AAV2‐sFLT01 AAV2‐hMERTK	[27] [28] [29]
AAV3	Primate	HSPG	FGFR1 HGFR LamR	Hepatocellular carcinoma, skeletal muscle, inner ear	scAAV3‐miR‐26a/122 AAV3B AAV3B‐V04 AAV3B‐DE5 AAV3‐ST (T492V + S663V)	[6] [30] [31] [32] [33]
AAV4	Primate	O‐linked sialic acid	Unknown	CNS, retina	AAV4‐hRPE65	[29]
AAV5	Human	N‐linked sialic acid	PDGFR	Skeletal muscle, lung, liver, CNS, retina	AAV5‐hPDE6B AAV5‐OPTIRPE65	[29]
AAV8	Primate	Unknown	LamR	Skeletal muscle, liver, pancreas, retina, heart, CNS	AAV8‐N94K AAV8‐W177R AAV8‐P307L AAV8‐R330Q AAV8‐G338E AAV8‐hCARp.Hcngb3 AAV8‐Hrlbp1 AAV8‐miR‐20a	[7] [34] [29]
AAV9	Primate	N‐linked galactose	LamR	Skeletal muscle, liver, heart, kidney, brain, lung, pancreas,	AAV9.452sub.LUNG1 AAV9‐PHP.B AAV9 TauP301L	[35] [36] [37]

The directed evolution strategy generates diverse AAV capsid libraries followed by artificial selection of variants with desired tropism, departing from existing proteins and AAV capsids, introducing mutations via various methods, and screening capsid progeny with enhanced traits, which has generated modified vectors for augmented delivery to different tissues [38, 39, 40, 41]. Novel myotropic AAVs named MyoAAVs and AAVMYOs have been discovered by a directed evolution approach, which share the integrin‐interacting Arginine, Glycine, Aspartic Acid (RGD) motif, have skeletal and cardiac muscle tropism, and liver de‐targeting effect [42, 43]. Among different myotropic AAV serotypes, MyoAAV2A exhibited the highest efficiency in transduction of leg muscle and heart, while MyoAAV4A and AAVMYO showed the highest efficiency in leg muscle and diaphragm transduction, respectively. Meanwhile, AAVMYO and MyoAAV4A presented better liver de‐targeting effect than MyoAAV2A [44]. Novel AAV5 capsid mutant AAVzk2 with robust liver tropism, which is generated by directed evolution and performed by library screening, exhibits high human hepatocellular transduction, low seroreactivity, and divergent post‐translational modification sites [45]. The engineered AAV9 variant, AAV9.452sub.LUNG1, which is derived by directed evolution of the surface‐exposed residues AA452‐458, exhibits high transgene expression in lung tissue, expands the toolbox for lung gene delivery, and displays promising application in lung‐specific preclinical research [35]. In the combinatorial AAV1 capsid library generated by capsid shuffle and directed evolution, AAV‐BBB6, AAV‐BBB28, and AAV‐BBB31 exhibited substantial improvement in the functional transduction of the central nervous system (CNS) with reduced liver tropism [26]. In the combinatorial AAV2 capsid library generated by rational design and directed evolution, P2‐V1(trpYF + TV) could efficiently evade neutralization and transduce NHP retina following intravitreal injection (IVtI) [27]. In the combinatorial AAV3B capsid library generated by rational design and directed evolution, AAV3B‐V04 is superior to AAV3B and AAV3B‐DE5 in the items of transduction efficiency, tissue tropism, vector immunogenicity, and gene transfer [30]. The combination of directed evolution with DNA‐family shuffled library and domain‐swapping strategies provides a genetic tool for generating vectors with improved human liver tropism, monitoring the evolution of key VRs for efficient human hepatocyte transduction, and identifying key capsid residues for enhanced primary human hepatocyte uptake [46].

### 2.2 Packaging Capacity

**Figure 3:**
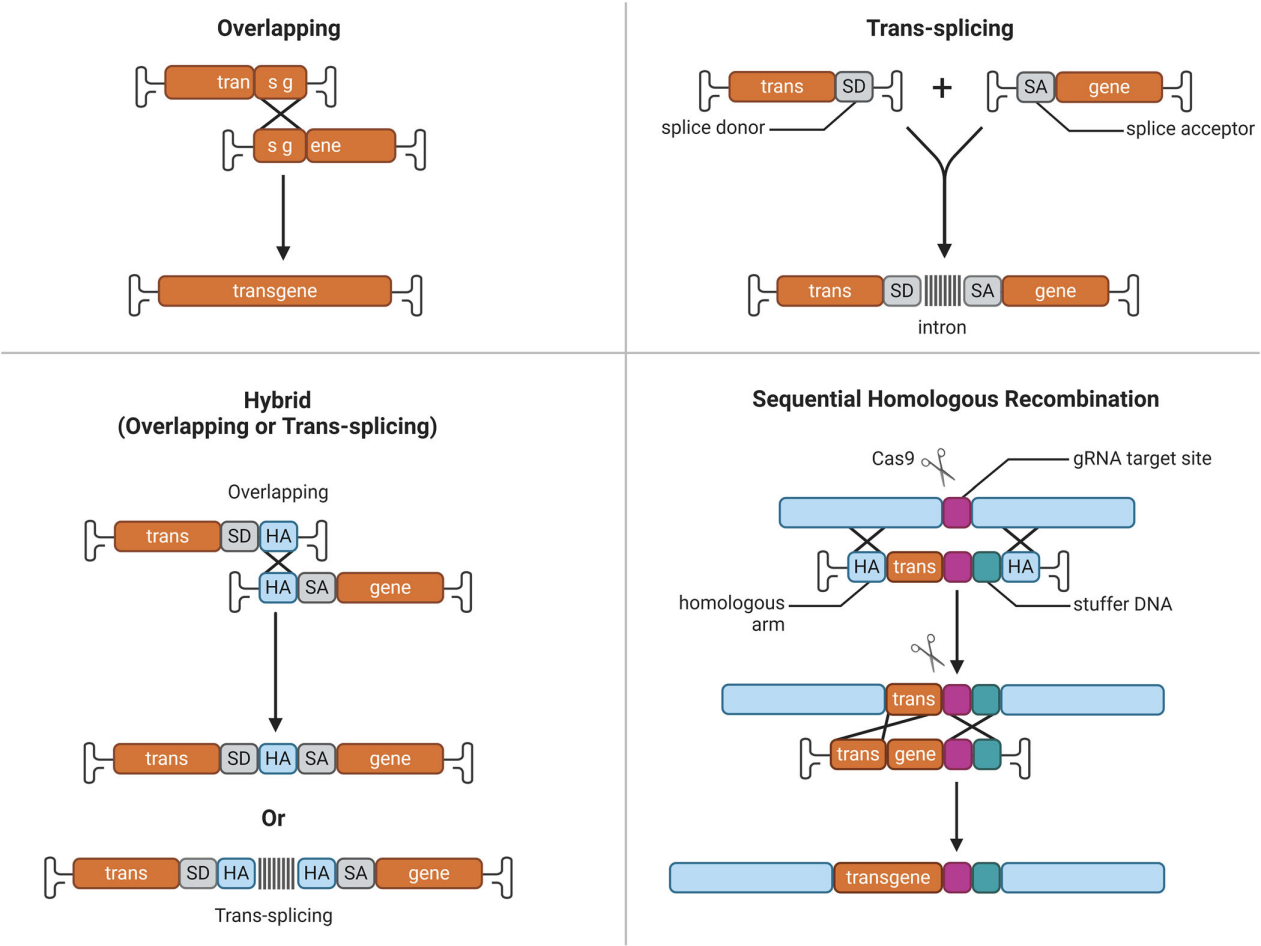
The strategies for enhancing AAV packaging capacity. To accommodate large transgenes, there are several strategies, such as overlapping, trans‐splicing, hybrid, and sequential homologous recombination.

The limited packaging capacity hinders the application of AAV for delivering large transgenes, cell‐type‐specific promoters, gene activators, and gene inhibitors in different tissues and organs [47, 48]. The optimum packaging size is between 4.1 and 4.9 kb, while oversized vectors (larger than 5.2 kb) exhibit reduced packaging capacity and transduction efficiency [49]. There are several strategies to expand AAV packaging capacity (Figure 3), such as expression cassette optimization, trans‐splicing, overlapping vectors system [50, 51, 52].

The expression cassette optimization could maximize packaging capacity with large transgene and high expression level by minimizing regulatory element size, splitting therapeutic protein, hybridizing overlapping segments, and inserting initial template such as enhanced green fluorescent protein (EGFP), woodchuck hepatitis posttranscriptional regulatory element (WPRE), and bovine growth hormone polyadenylation signal (bGHpA) [53, 54, 55]. The insect cell‐based baculovirus expression vector (BEV) system is a well‐established leading platform for AAV scalable production, such as the first regulatory‐approved AAV gene therapy drug (Glybera) [56]. The optimized *Rep* gene expression is achieved by the application of alternate, non‐conservative baculovirus promoters (p10, 39k, p6.9, and pSel120) with distinct expression intensities and temporal profiles, which exhibit improved In Vitro potency, high AAV particles yield, reduced promoter‐promoter competition, and low toxic protein expression [57]. The optimized transgene expression cassette for human coagulation factor IX (hFIX) is based on AAV3 serotype with the strongest tropism for human hepatocytes among the commonly used serotypes [31, 58]. The progress is further enhanced by several steps, such as engineered AAV3 variant AAV3‐ST (T492V + S663V), insertion of hepatocyte‐specific enhancer and coding region, depletion of CpG motifs [32, 33, 59].

The trans‐splicing approach reconstitutes gene expression from two independent AAV vectors, which requires intermolecular concatamerization and subsequent splicing between independent vectors [60]. The trans‐splicing‐mediated AAV gene therapy could be classified into several steps: dual‐vector coinfection into the same cell, head‐to‐tail recombination among incoming vector genomes, transcription elongation through the ITR junction and pre‐mRNA stability, and splicing across the ITR junction [61, 62]. There are several factors, such as viral genome recombination, virus infection dose, two independent AAV vectors coinfection, pre‐mRNA stability, mRNA accumulation, as well as transcription and splicing across the ITR junction, affecting trans‐splicing approach efficiency by inhibiting the transcription of heterogeneous nuclear RNA and increasing the probability of intermolecular concatamerization [63]. The overlapping vector approach splits the gene into two partially overlapping fragments separately, packages the fragments into upstream and downstream vectors, respectively, utilizes homologous recombination during overlapping regions in two independent vectors to reconstitute full‐length messenger and correct the histopathology [64, 65]. The overlapping vector approach is widely used in transferring either β‐galactosidase or minidystrophin to skeletal muscle following both intramuscular and intravenous injection, as well as transferring eye‐disease–related genes, MYO7A or ABCA4, to the retina following subretinal injection [66, 67, 68, 69]. Compared with the trans‐splicing approach, the overlapping vector approach is much easier to manipulate without the requirements of complicated cloning procedures [70].

The breakthrough dual/triple vector approaches were achieved by the combination of trans‐splicing and an overlapping vector to split large transgenes into two or three fragments and package them into separate AAV capsids [71, 72, 73]. Dual‐vector approach based on intein‐mediated protein recombination using synthetic AAV9‐PHP.B vectors could circumvent the size limitation of AAV vectors, restore STRC protein expression, and replace full‐length wild‐type *Strc* in outer hair cells of *Strc*‐mutated DFNB16 mice [36, 74]. The dual‐AAV split prime editor (split‐PE) system with two efficient split‐PEs (split‐PE1005 and split‐PE1024) reconstructed by Rma intein using EGFP‐based reporter cells could pave the way for In Vivo gene‐editing therapy using PE [75]. Dual‐AAV‐ie vector groups, dual AAV‐ie‐CAG vectors (AAV‐ie‐CAG‐EGFP and AAV‐ie‐CAG‐mCherry), and dual‐AAV‐ie‐CMV vectors (AAV‐ie‐CMV‐EGFP and AAV‐ie‐CMV‐mCherry), achieve efficient co‐transduction in the neonatal and adult mouse utricles, which are affected by cellular entry, intracellular events, interaction between the promoters and RNA polymerase II [76]. The flexible dual AAV vector technology based on reconstitution via mRNA trans‐splicing (REVeRT) could functionally and efficiently reconstitute split genes, coding sequences, CRISPRa module, and full‐length ABCA4 [77].

### 2.3 Immune Response

The viral capsid proteins with inherent immunogenicity could trigger pre‐existing and therapy‐induced immune responses, which could impact safety, efficacy, and potential for AAV‐mediated gene therapy [78]. The immune response is classified into two categories: innate and adaptive [79]. Innate immune response, the first line of defense against pathogenic substances without limitation to a single pathogenic agent, could activate all three main pattern recognition pathways (Toll‐like, NOD‐like, and RIG‐I‐like receptor pathways) and initiate Th1 response [80]. Adaptive immune responses, which consist of humoral and cellular immune responses, are affected by many factors such as administration route, vector design, target gene expression, and target organ disease [81].

**Figure 4:**
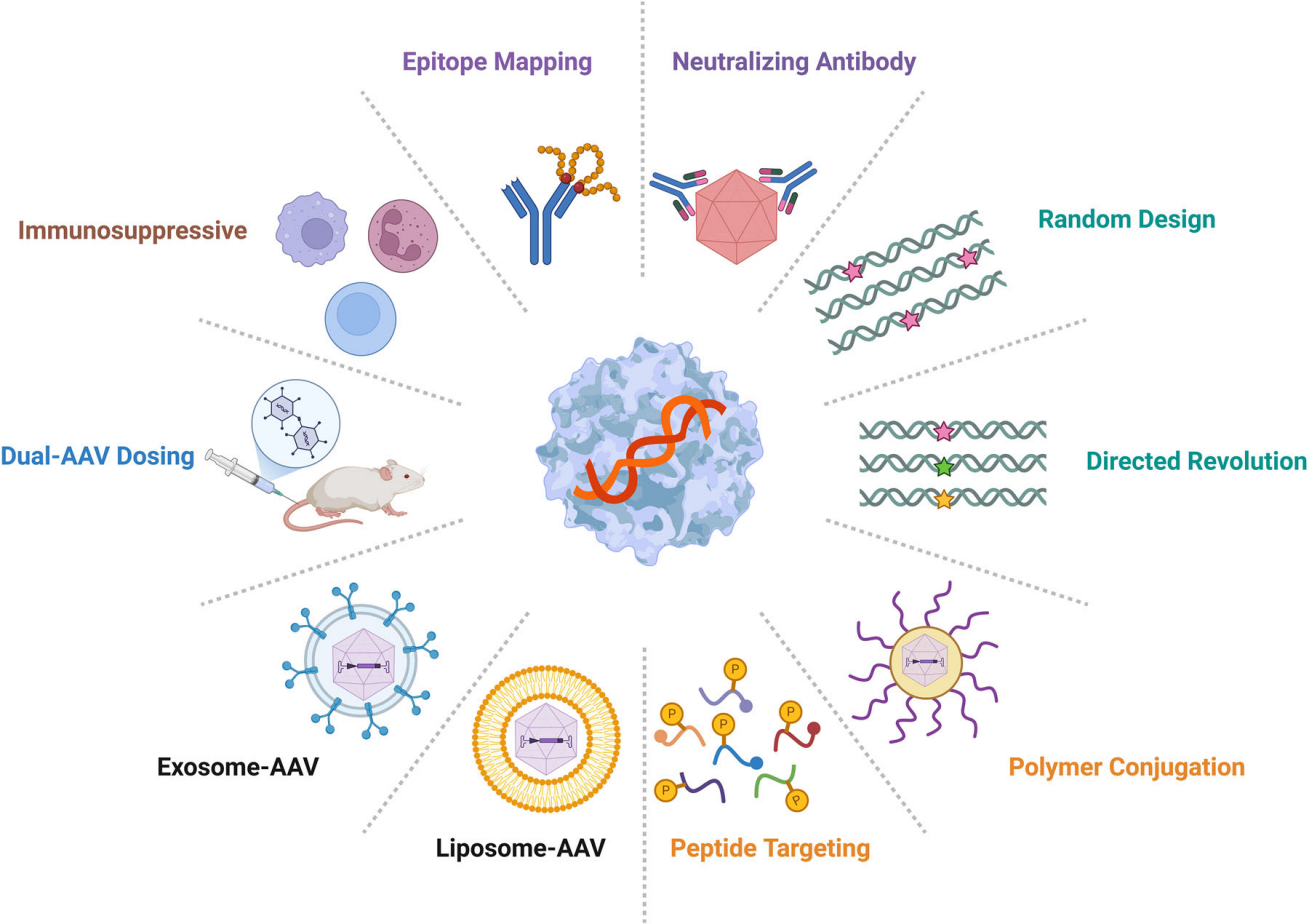
The strategies for reducing AAV immune response. The host antiviral immune responses directed against AAV capsid and transgene product could be resolved by several strategies, such as capsid engineering (rational design, directed revolution), surface tethering (polymer conjugation, peptide targeting), viral load (liposome‐AAV, exosome‐AAV), dual‐AAV dosing, immunosuppressive, molecular imprinting (epitope mapping, neutralizing antibody).

Humoral immune responses to AAV include two types: neutralizing antibodies (NAbs) inhibiting AAV transduction, and non‐neutralizing antibodies (non‐NAbs) without blocking AAV transduction [82]. Cellular immune responses to AAV are capsid‐specific and dose‐dependent, which include T cells, macrophages, and cytokines [83]. The innate immune system has a critical function for mediating the initial vector response and priming the adaptive immune system [84]. There are several methods for modifying and modeling immune responses (Figure 4), such as capsid engineering, surface tethering, viral load, dual‐AAV dosing, immunosuppressive, and molecular imprinting [85, 86, 87].

The capsid engineering of AAV2 by inserting MyD88‐derived peptide RDVLPGT to generate a novel capsid variant AAV2.MB453, without interfering with capsid assembly and production yield, could reduce innate immune responses In Vitro and attenuate adaptive immune responses In Vivo [28]. The surface tethering strategies, such as polyethylene glycol tethering (PEGylation), utilize chemical modifications to evade NAbs or achieve tissue targeting, which affect cell interaction and internalization as well as tissue accumulation and retention [88]. The viral load strategies, noncovalent surface modification strategies, take advantage of pharmaceutical methods (liposomes, exosomes, hydrogels, microneedles) to modify natural targets, change AAV tropism, and increase transduction efficiency in liver, lung, muscle, ear, and retina [89, 90, 91, 92]. The molecular imprinting therapy investigates AAV‐Nabs binding to identify specific epitopes and hypervariable regions, which could be widely used for the prevention of AAV's neutralization [93]. The dual‐AAV dosing approach takes advantage of reduced initial priming dose to accurately reflect naturally acquired AAV immunity, which reveal the activation of classical complement pathway (TNF signaling, TLR signaling, and NF‐κB signaling) and identify myeloid‐derived chemokines/cytokines (TNF‐α, CXCL2, CCL3, CCL4, CCL5, CXCL10, IL‐1α, and IL‐1β) of both innate and adaptive immunity [94]. The immunosuppressive agents, such as corticosteroids, rapamycin, mycophenolate mofetil, tacrolimus, rituximab, eculizumab, and hydroxychloroquine, are used in AAV‐mediated gene therapy to target the CD4‐mediated helper function and reduce circulating IgG and plasma cell viability [95, 96].

### 2.4 Artificial Intelligence

AI have rapidly developed due to abundant mathematical tools and computational resources, which is clarified into ML (supervised learning, unsupervised learning, and reinforcement learning) and deep learning (DeepAffinity and DeepDTAF), which was awarded jointly to John Hopfield and Geoffrey Hinton by the Nobel Prize in Physics 2024 [97, 98, 99, 100, 101]. AI approaches are applied in AAV‐mediated gene therapy, such as logistic regression and convolutional/recurrent neural networks, as well as AAV capsid and library design towards capsid viability and genome assembly, which could increase on‐target transduction, reduce off‐target effect, advance viral immunology, decrease manufacturing costs, and enable closed‐loop and de‐immunized capsids engineering [102, 103].

Compared with rational design and directed evolution, ML mitigates the trade‐off between yield (fraction of successful samples generated per attempt) and throughput (number of samples), which opens new avenues of discovery through high‐throughput direct synthesis [104]. In supervised training schemes, ML algorithms learn arbitrary sequence‐to‐function relationships automatically from large datasets of capsid sequences and their measured properties, viable capsid, and tissue tropism [105, 106]. ML methods often take advantage of internal latent representations, such as principal component analysis (PCA), and could predict the emergence of escape mutations in multiple viruses [107]. ML process, known as transfer learning, transfers information across cell types and experimental contexts which is based on In Vitro capsid performance in diverse cell transduction experiments, and predicts In Vivo transduction results in the brain neurons which is used to refine model performance and understand the relationship between In Vivo and In Vitro assays [108, 109]. Meanwhile, transfer learning can integrate information from multiple modalities, which could accelerate the application of ML models in limited data areas for predicting and designing immune‐evasive capsids [110]. ML models are used at multiple steps of capsid engineering to generate AAV capsid libraries with high diversity, with large mutation numbers and retained packaging viability [111, 112]. Compared with standard libraries used today, ML‐guided AAV capsid libraries exhibited higher packaging fitness and variant number, which could be applied in individual gene sequence specification, predictive fitness model application, designed library diversity control, and various library construction applications [113]. Fit4Function, an ML‐based approach for systematically engineering multi‐trait AAV capsids (liver‐targeted and manufacturable), could predict cross‐species traits and conduct biodistribution assessment of peptide‐modified AAV capsids [114].

## 3 AAV in Cancer Drug Discovery

**Figure 5:**
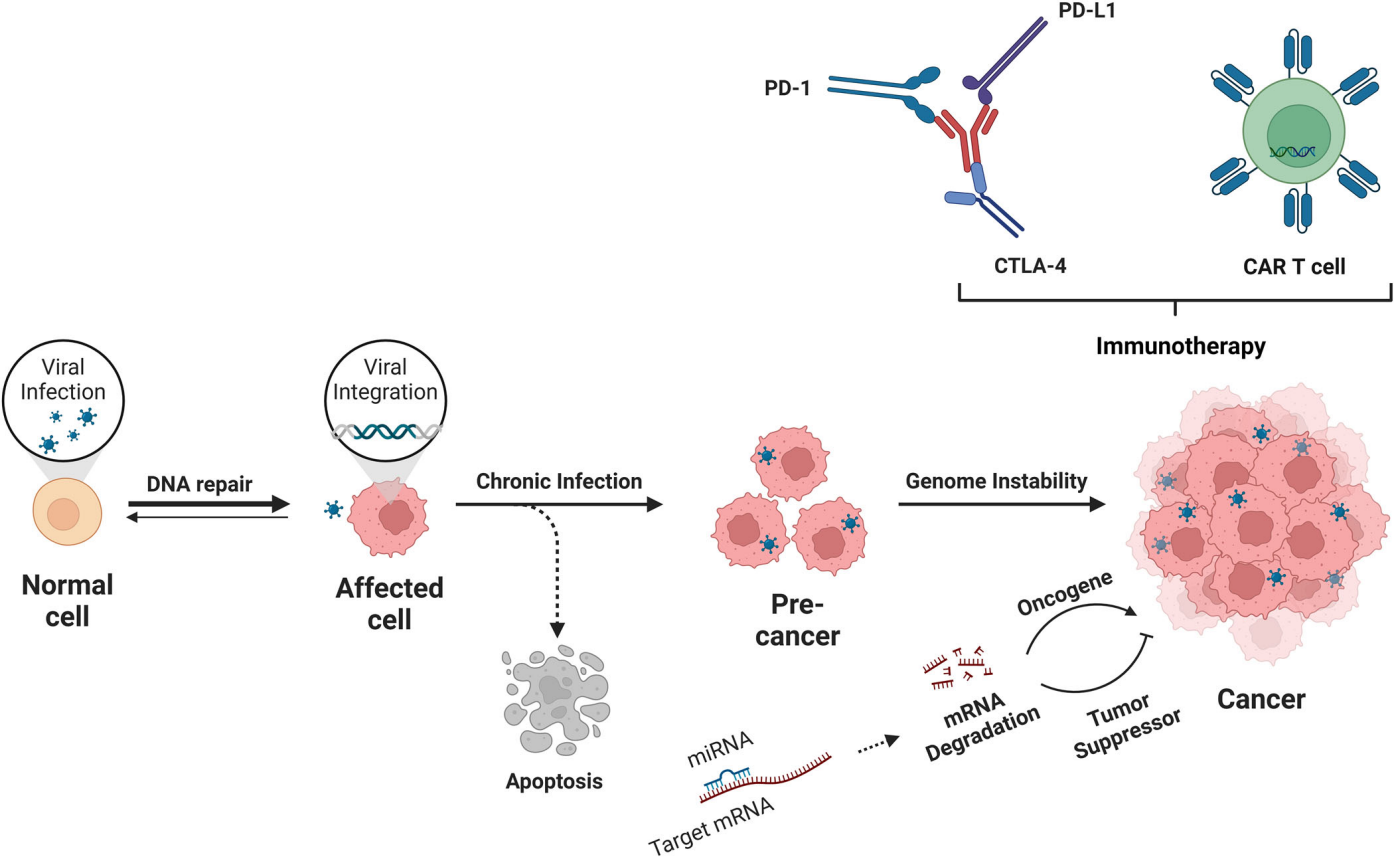
Combination therapy of AAV with miRNA/ICI/ACT could boost the cancer drug discovery by inhibiting tumor initiation, growth, and metastasis. The figure illustrates key pathways in cancer development: viral infection outcomes in normal vs. affected cells (DNA repair vs. integration), genomic instability in precancerous states with mRNA targets, oncogenic transformation with mRNA degradation, immune checkpoint markers, and CAR T‐cell immunotherapy.

As the most devastating diseases in the world, in the U.S. alone, more than 1.5 million cancer cases are diagnosed each year, with a relative 5‐year survival rate of 68% according to the Centers for Disease Control and Prevention (CDC) [115, 116]. The cancer hallmarks comprise biological capabilities acquired during the multistep tumor development and serve as blueprint for the development of new treatment strategies, which include evading growth suppression (cyclin‐dependent kinase inhibitors), avoiding immune destruction (immune activating anti‐CTLA4 mAb), enabling replicative immortality (telomerase inhibitors), tumor promoting inflammation (selective anti‐inflammatory drugs), activating invasion and metastasis (HGF/c‐Met inhibitors), inducing angiogenesis (VEGF inhibitors), genome instability and mutation (PARP inhibitors), resisting cell death (proapoptotic BH3 mimetics), deregulating cellular energetics (aerobic glycolysis inhibitors), sustaining proliferative signaling (EGFR inhibitors) [117, 118, 119]. There are several therapeutic approaches for cancer treatment, such as surgery, thermotherapy, chemotherapy, radiotherapy, immunotherapy, and gene therapy [120, 121, 122]. Due to the characteristics of high transferring ability, low host immune response, and long‐term gene expression, in combination with several technologies (miRNA, ICI, ACT), AAV have been successfully used for cancer gene therapy to deliver and transfer therapeutic genes (suicide genes, anti‐angiogenic genes, and immune‐related genes) to cancer cells for inhibiting tumor initiation, growth, and metastasis (Figure 5).

MicroRNAs (miRNAs), a class of small, endogenous, single‐stranded, noncoding RNAs, play a major role in viral life cycle, infectivity, and pathogenicity [123]. HCC, the most common type of liver cancer and the fourth leading cause of cancer deaths worldwide, is a highly vascularized tumor with a high 5‐year postoperative recurrence rate and poor 5‐year survival rate [124, 125]. MicroRNA‐342‐3p (miR‐342‐3p) serves as a tumor suppressor in HCC through inhibition of IGF‐1R expression, glucose uptake, and ATP production [126]. In Vivo administration of AAV‐miR‐342‐3p attenuates tumor development and prolongs the survival of LT2/MYC and LT2/RAS mice [127]. Both miRNA 122a and miRNA199a exhibited reduced expression in most HCC cell lines Hep3B, PLC/PRF/5, SKHep1, and SNU423. The dual‐regulated gene delivery system, AAV8‐miR122a‐miR199a, regulates transgene expression and harbors binding sites [128]. Both miRNA26a and miRNA122 act as liver‐specific miRNA to regulate proliferation, apoptosis, metastasis, angiogenesis by targeting IL‐6‐Stat3, PIK3C2α/Akt/HIF‐1α/VEGFA, HGF‐c‐Met, Wnt/β‐catenin, cyclin G1/E2F1, and TCF‐4 [129, 130, 131, 132]. The dual HCC‐targeted construct, AAV3‐miR26a‐miR122, leads to ed to ~26% and ~70% inhibition of HCC growth In Vitro and In Vivo [6]. MicroRNA‐20a (miR‐20a) serves as a key effector of ATP synthesis and apoptosis to affect the metabolic and apoptotic functions of cytochrome c (CYCS) [133]. The transgenic HCC mouse models, MYC/LT2 mice, show clear attenuation of tumor development via AAV‐mediated miR‐20a‐Tough‐Decoy treatment [34].

Immune checkpoint inhibitors (ICIs), with the localization on T cells, B cells, and dendritic cells (DCs), can block distinct receptors on T cells or tumor cells and prevent T cell inactivation and tumor immune escape, which include programmed cell death 1 (PD‐1; targeted by Nivolumab, Pembrolizumab, Cemiplimab), programmed cell death ligand 1 (PD‐L1; targeted by Atezolimumab, Durvalumab, Avelumab), and cytotoxic T‐lymphocyte antigen 4 (CTLA4; targeted by Ipilimumab, Tremelimumab) [134, 135, 136]. PD‐1, together with its ligands PD‐L1/PD‐L2, play critical roles in tumor evasion, pathway blockade, and cytotoxic activity to mediate antitumor therapy. The anti‐tumor AAV‐mediated delivery of soluble extracellular domain of PD‐1 (sPD‐1) could reduce tumor microenvironment inhibitory effects on T cells and enhance cytotoxicity by lymphocytes stimulated specifically with an antigen [137]. The sPD1‐based DNA vaccination strategy is based on DC, which cross‐primes antigen‐specific CD8^+^ CTLs with potential for tumor immunotherapy [138]. AAV‐DJ‐sPD1‐TWIST1 fusion vaccine elicits two types of antitumor effects: enhances antigen‐specific T cell responses and immune checkpoint blockade, arrests and eradicates established malignant mesothelioma [139]. Her2‐AAV, which displays Her2/neu‐specific DARPins on the capsid surface, could enable specific gene transfer in subcutaneous and disseminated Her2/neu+ positive tumor lesions in a xenograft tumor mouse model [140]. Two engineered AAV vectors, self‐complementary (sc) AAV vectors encoding murine αPD‐1 in the scFv‐Fc format (HER2‐AAV‐IgG‐Fc) and single‐stranded (ss) AAV vectors encoding the full‐length antibody nivolumab (HER2‐AAV‐αPD‐1), lead to efficient tumor cells transduction, functional aPD‐1 secretion and subsequent T cells reactivation without affecting target antigen expression, which could be applied in straight‐forward tumor‐targeted ICI delivery in combination with chemo‐ or radiotherapy [141, 142].

Adoptive cell transfer (ACT) serves as burgeoning therapeutic form of human immuno‐oncology for engrafting exogenous lymphocytes into recipient host to combat disease, which includes tumor‐infiltrating lymphocyte (TIL) therapy, engineered T cell receptor (TCR)‐T cell therapy, chimeric antigen receptor (CAR)‐T cell therapy [143, 144]. CAR T‐cell therapy, with T cell sources from the patient (autologous) and donor (allogenic), received marketing approval from the FDA for children with acute lymphoblastic leukemia and adults with advanced lymphomas [145, 146]. AAV‐Cpf1 system generated by the combination of mRNA electroporation for LbCpf1 and AAV, could build stable, efficient and one‐step CAR‐T (AAV‐Cpf1 KIKO CAR‐T) with homology‐directed repair knockin and checkpoint knockout, which could serve as T cell engineering research tool for improved “off‐the‐shelf” adoptive T cell therapies in the clinic with multiple advantageous features such as CAR retention, cancer cell killing, effector function and resistance to exhaustion [147]. The AAV‐derived closed‐ended linear duplex DNA (CELiD) vector, which integrates CAR genes into the AAVS1 site, could increase cytokine secretion and remove safety constraints of CAR‐T therapy [148]. The novel approach AAV delivering CAR gene therapy (ACG) with plasmid DNA encoding a CD4‐CAR, causes effective tumor regression and produces antitumor immunological characteristics, which could skip the technical ex vivo procedures challenges and induce the host to create its CAR T cells [149].

## 4 AAV in Neurodegenerative Disease Drug Discovery

**Figure 6:**
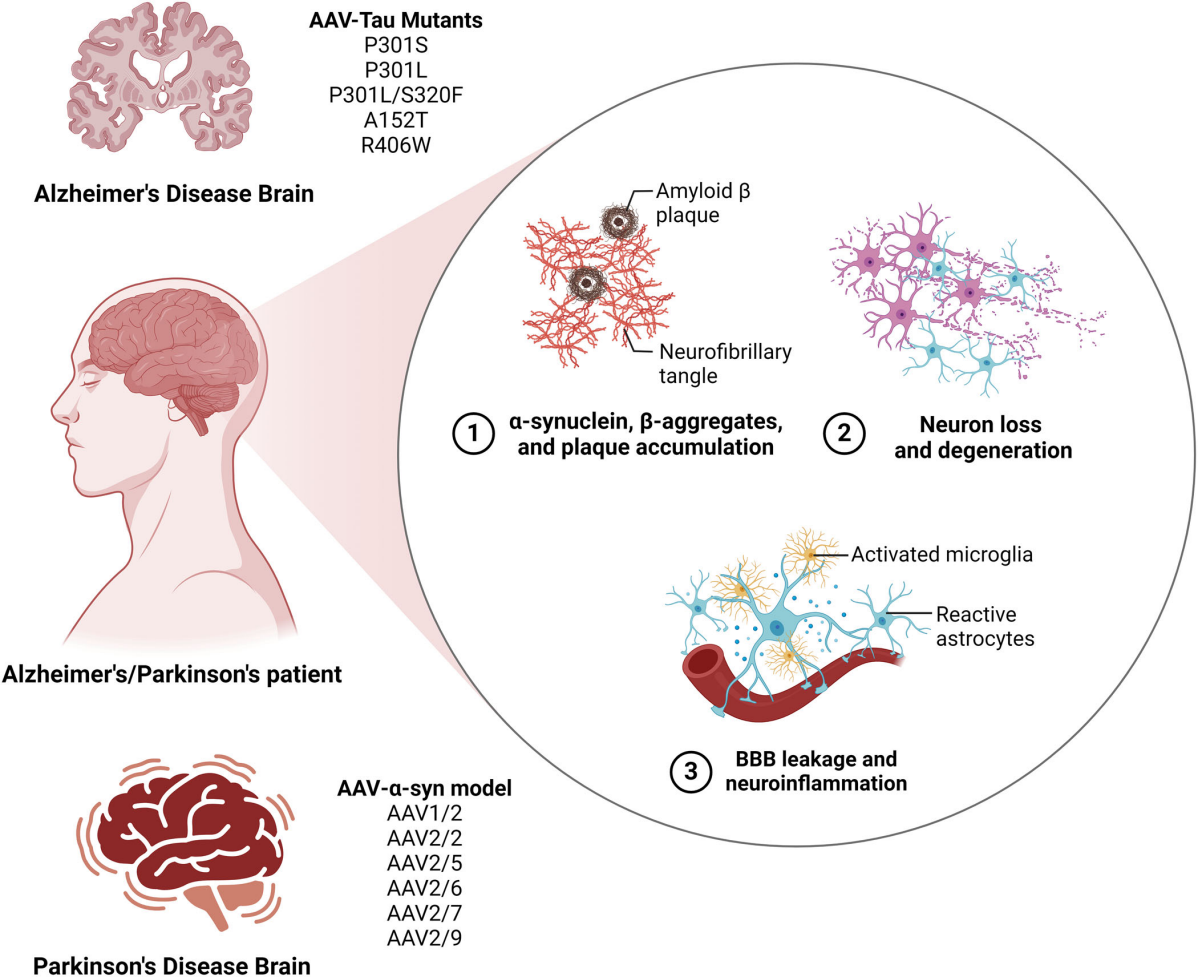
AAV‐mediated gene therapy could treat neurodegenerative diseases by overcoming blood–brain barrier, reprogramming capsid proteins, and selecting various serotypes. This figure illustrates the use of AAV vectors to model neurodegenerative disorders, highlighting key tau mutants (P301S, P301L, P301L/S320F, A152T, R406W) associated with Alzheimer's pathology, along with characteristic neuropathological features including amyloid β plaques, neurofibrillary tangles, α‐synuclein aggregates, and associated neurodegeneration. It further depicts neuroinflammatory components (activated microglia, reactive astrocytes, BBB leakage) in Alzheimer's/Parkinson's patients, and presents various AAV serotypes (AAV1/2 to AAV2/9) employed for modeling α‐synuclein pathology in Parkinson's disease.

Neurodegenerative diseases are debilitating disorders with different pathological patterns and clinical manifestations, which include AD, Parkinson's disease (PD), Huntington's disease (HD), SMA, and amyotrophic lateral sclerosis (ALS) [150, 151, 152]. AAV‐mediated gene therapy paves the way to treat neurodegenerative diseases by multiple strategies (Figure 6), such as revolutionizing disease mechanisms understanding, overcoming blood–brain barrier (BBB), as well as reprogramming capsid proteins and various serotypes [153, 154, 155]. In recent years, AAV‐mediated gene therapy has gained clinical interest for treating SMA (NCT02122952; NCT03306277, NCT03381729), AD (NCT03634007) [156].

AD is the most common age‐related neurodegenerative disease with two major hallmarks (amyloid‐β and tau protein aggregates), which is associated with progressive cognitive deterioration, such as memory impairments and cognitive decline [157, 158, 159]. AAV‐tau injected mice had pathological features (tau conformational changes, neuronal loss, glial activation, cortical atrophy) as well as behavioral changes (abnormal open field, increased anxiety, reduced learning, and memory) [37, 160]. AAV microinjection of mutant tau (Tau‐P301L) to forebrain organoids induces pronounced tau aggregates containing tau fibrils, which could overcome the limitation of tauopathy recapitulation in the very early disease stage [161]. AAV‐mediated gene therapy of double tau mutation (Tau‐P301L/S320F) in young adult rhesus monkey generates profound tau‐based neuropathology, induces misfolded tau propagation and accelerates intense neuroinflammation, with reflections in the cerebrospinal fluid and plasma [162]. Compared with Tau‐P301L, AAV‐Tau‐A152T injected mice exhibit increased tau phosphorylation and soluble pT153 levels [163]. Compared with Tau‐P301L, AAV‐Tau‐R406W injected mice yield a lower amount of tau oligomers with high Htr7, Htr1a, and Cdk5 expression as well as low Bdnf and Ntrk2 expression [164, 165]. AAV‐mediated gene therapy of c‐terminal cleaved tau at D421 (∆D421‐tau) leads to significant neuronal loss in the CA3 area of the hippocampus and medial entorhinal cortex, which contributes to synaptic plasticity and cognitive deficits [166]. The administration of AAV‐vectored anti‐phospho‐tau antibody PHF1 to P301S tau transgenic mice exhibits reduced hippocampal tau pathology, decreased insoluble pathological p‐tau species and NFTs, durable hippocampal antibody levels, as well as abundant intraneuronal antibody expression [167].

PD is the most common neurodegenerative movement disorder with two major hallmarks (degeneration of dopaminergic neurons within the substantia nigra *pars compacta* (SNpc) as well as formation of α‐synuclein‐containing protein aggregates Lewy‐bodies and Lewy neurites), which is characterized by parkinsonian motor deficits and numerous non‐motor symptoms [168, 169, 170]. AAV‐α‐syn model has become widely used for inducing and modeling PD‐like synuclein pathology in different targets, such as neurons (brainstem noradrenergic and serotonergic neurons) and oligodendrocytes (prime synucleinopathy target in multiple system atrophy), which is initially in rats, and later also in mice and NHPs [171, 172, 173]. AAV‐α‐synuclein based rodent models drive α‐syn transgene and synuclein pathology by the application of different AAV serotypes and cell type‐specific promoters, which includes single hit models using the AAV‐α‐synuclein vector alone (substantia nigra, locus coeruleus, midbrain raphe nuclei, basal forebrain nuclei, striatum) as well as double hit models in combination with a second, interacting risk factor (Rotenone, impaired GBA1, LRRK2 mutation) [174, 175, 176, 177, 178, 179].

There are several serotypes involved in the AAV‐α‐syn model, such as AAV1/2, AAV2/2, AAV2/5, AAV2/6, AAV2/7, AAV2/9, which work well in rats compared with those in mice, in terms of TH+ cell loss and behavioral impairments [180, 181, 182, 183]. The optimized promoter and enhancer constructs involved in the AAV‐α‐syn model, such as WPRE, CMV, CBA/CMV hybrid, Synapsin‐1, CBAie‐enhanced Synapsin‐1, can visualize and quantify α‐syn delivery [184, 185]. The working titers of the AAV‐α‐syn model are adjusted at a high level for advanced, symptomatic disease as well as at a moderate level for early, pre‐symptomatic disease [186, 187].

## 5 AAV in Retinal Disease Drug Discovery

**Figure 7:**
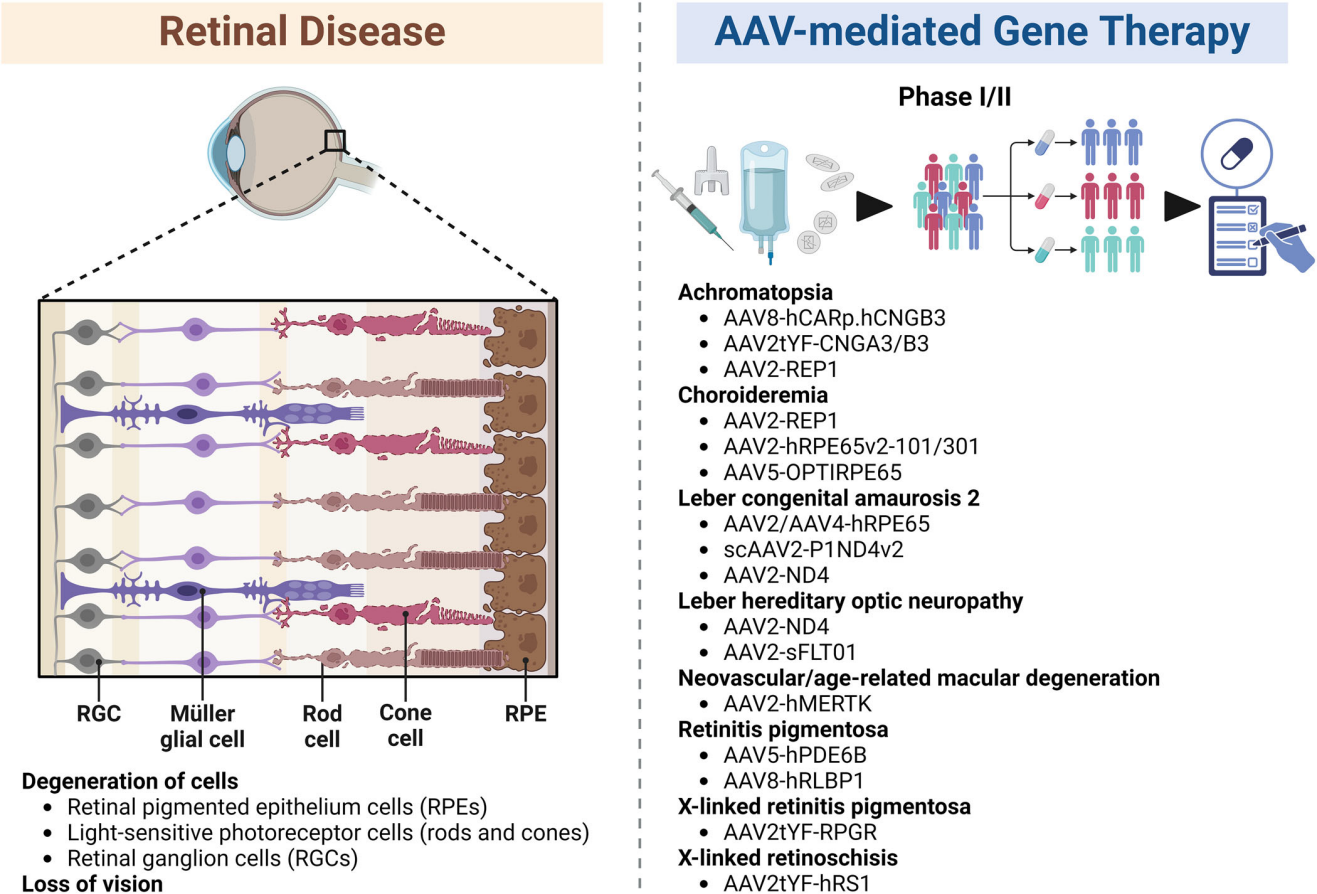
AAV‐based gene therapy clinical applications for retinal diseases. There are several breakthroughs in retinal diseases, such as Achromatopsia, Choroideremia, Leber congenital amaurosis 2, Leber hereditary optic neuropathy, Neovascular/age‐related macular degeneration, Retinitis pigmentosa, X‐linked retinitis pigmentosa, and X‐linked retinoschisis, demonstrating the versatility of AAV serotypes (AAV2, AAV5, AAV8) for ocular gene delivery.

AAV‐mediated gene therapy for retinal diseases reached Phase I/II clinical trials (Figure 7), which targets diseases such as Achromatopsia (AAV8‐hCARp.hCNGB3, AAV2tYF‐CNGA3/B3, AAV2‐REP1), Choroideremia (AAV2‐REP1, AAV2‐hRPE65v2‐101/301, AAV5‐OPTIRPE65), Leber congenital amaurosis 2 (AAV2/AAV4‐hRPE65, scAAV2‐P1ND4v2, AAV2‐ND4), Leber hereditary optic neuropathy (AAV2‐ND4, AAV2‐sFLT01), Neovascular/age‐related macular degeneration (AAV2‐hMERTK), Retinitis pigmentosa (AAV5‐hPDE6B, AAV8‐hRLBP1), X‐linked retinitis pigmentosa (AAV2tYF‐RPGR), X‐linked retinoschisis (AAV2tYF‐hRS1) [29, 188, 189]. The major breakthrough in retinal gene therapy was realized in 2017 for FDA approval of AAV vector Luxturna‐mediated gene therapy for Leber congenital amaurosis type 2 (LCA2), which is caused by RPE65 mutations leading to severely impaired vision at birth and then slowly progressive degeneration of retinal photoreceptors [190].

The clustered regularly interspaced short palindromic repeats (CRISPR) and CRISPR‐associated (Cas) protein 9 system (known as CRISPR/Cas9) is a powerful genome‐editing system consisting of the Cas9 nuclease and modified single guide RNA (sgRNA), which have been applied in AAV‐mediated gene therapy and editing in the retina [191, 192]. Gain‐of‐function mutations in retinal guanylate cyclase 2D (GUCY2D), which encodes retinal guanylate cyclase 1 (RetGC1), are the leading cause of cone‐rod dystrophy 6 (CORD6) with the loss of early‐onset cone cell and central/color vision [193]. AAV‐CRISPR‐Cas9‐based approach delivers CRISPR‐Cas9‐targeted wild‐type/mutant GUCY2D alleles coding sequence as well as CRISPR‐Cas9‐resistant GUCY2D cDNA copy for knocking out endogenous RetGC1 expression and supplementing exogenous GUCY2D healthy copy, which stably preserves retinal structure and function in a novel CORD6knockin mouse model (RetGC1) [194]. Heterozygous variants in the *RHO* gene encoding for rhodopsin (RHO), a visual 348 amino acid G‐protein‐coupled receptor, are the leading cause of autosomal dominant retinitis pigmentosa (RP), which is a group of inherited retinal degenerative diseases due to loss of rod/cone photoreceptors [195]. The combination of a high‐fidelity Cas9 variant carrying seven amino acid substitutions, Asn497Ala, Arg661Ala, Gln695Ala, Gln926Ala, Asp1135Val, Arg1335Gln, and Thr1337Arg, with allele‐specific gRNAs to generate p.Pro347Sser RHO, leads to partial recovery of photoreceptor function [196]. Mutations in highly conserved PRPF31, pre‐mRNA processing factor 31, composed of 14 exons, 1 non‐coding and 13 coding, account for 6%–11.1% of autosomal dominant RP [197]. AAV‐CRISPR/Cas9‐PRPF31 constructs mediated efficient PRPF31 knockout and restored the retinal structure, normal phagocytosis, cilia formation, and barrier function [198]. Variants within the Retinitis Pigmentosa GTPase regulator (RPGR) gene, which is characterized by progressive degeneration and photoreceptors loss, are the predominant cause of X‐Linked Retinitis Pigmentosa (XLRP) [199]. AAV‐CRISPR/Cas9‐RPGR approach could successfully restore RPGR mRNA and protein expression, glutamylation, and rhodopsin localization, which provide mechanistic insights into vector potency in human photoreceptor cells [200].

## 6 AAV in SARS‐CoV‐2 Vaccine

SARS‐CoV‐2 is a highly infectious respiratory disease, which shows a high infection rate, a long incubation period, and rapidly emerging variants [201, 202, 203]. As of July 2024, SARS‐CoV‐2 has caused 775 million infections and over 7 million deaths worldwide [204]. There are rapid mutation accumulations of SARS‐CoV‐2 variants, which includes Alpha (B.1.1.7), Beta (B.1.351), Gamma (P.1), Kappa (B.1.617.1), Delta (B.1.617.2 and AY.1/2), Epsilon (B.1.427 and B.1.429), Omicron (B.1.1.529), Eta (B.1.525), Iota (B.1.526), Lambda (C.37), Mu (B.1.621), and Theta (P.3) [205, 206, 207]. Currently, there are more than 150 vaccines in clinical trials and nearly 200 pre‐clinical vaccine candidates, which include inactivated or weakened virus vaccines (CoronaVac, BBIBP‐CorV, Covaxin), protein‐based vaccines (NVX‐CoV2373, ZF2001, V‐01, SCB‐2019, Bimervax, SCTV01C, SCTV01E, and SCTV01E‐2), viral vector vaccines (Vaxzevria, JNJ‐78436735, Sputnik V, Sputnik light, CONVIDECIA), and RNA and DNA vaccines (COVIGEN, AG0302, GX‐19N, Spikevax, Comirnaty, HDT‐301) [208, 209, 210, 211]. According to the World Health Organization (WHO) COVID‐19 Vaccination Insights Report from Jan 2024 to Mar 2024, 9.8 million individuals have received a dose of the COVID‐19 vaccine across 73 reporting Member States, containing 22% of the global population [204].

**Figure 8:**
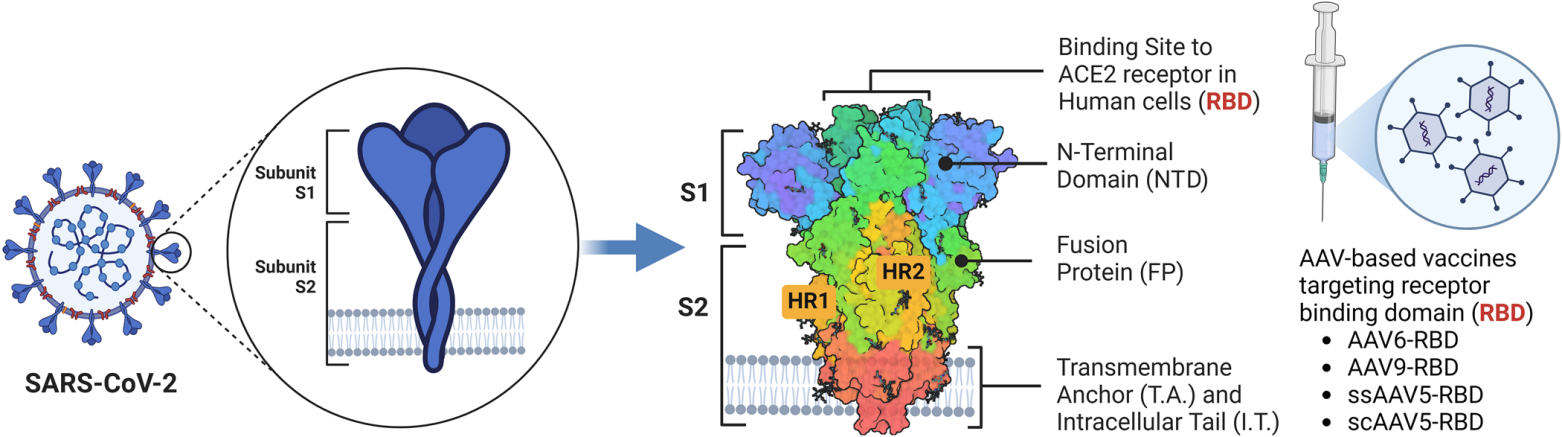
AAV‐based COVID‐19 vaccines target SARS‐CoV2 S protein RBD. The figure illustrates key functional domains of the SARS‐CoV‐2 spike protein, including the receptor binding domain (RBD) that interacts with human ACE2 receptors and the N‐terminal domain (NTD). Also depicted are AAV‐based vaccines targeting RBD, including AAV6‐RBD, AAV9‐RBD, ssAAV5‐RBD, and scAAV5‐RBD.

AAV‐based COVID‐19 vaccines (Figure 8) elicit low B and T cell immunogenic responses and longer‐lasting gene expression with thermostability and manufacturing potential [212, 213]. AVCOVID, an AAV‐ and gene‐based spike antigen COVID‐19 vaccine, demonstrates viral protection, durable immunogenicity, variant cross‐reactivity, low‐dose efficacy, and room‐temperature stability in mice and NHPs [214, 215, 216]. AAV‐based vaccines targeting RBD of the SARS‐CoV‐2 S protein, including AAV‐RBD (max), AAV‐RBD (wt), AAV‐2xRBD, and AAV‐3xRBD, elicit strong and long‐lived immune responses, comparable cross‐protection as well as powerful NAbs against SARS‐CoV‐2 infection [217]. S663V‐RBD, an AAV6‐ and capsid and antigen structure engineering (CASE)‐based vaccine fused with biological adjuvant RS09, could rapidly induce satisfactory RBD‐specific IgG titer, endurable and robust immune responses, high safety profile and storage stability, NAbs against three typical SARS‐CoV‐2 pseudoviruses (wildtype, Lambda, and Delta) [218]. AAV9‐RBD, a virus vaccine with two linked RBDs, could efficiently elicit immune responses, produce long‐lasting antibodies, offer long‐term protection, and infect both muscle and airway epithelial cells against SARS‐CoV‐2 [219]. Single‐stranded (ssAAV5)‐RBD‐plus vaccine induced long‐term humoral‐mediated immune and specific T‐cell responses as well as high levels of RBD‐specific IgGs, provided substantial protection, and exhibited NAbs against three typical SARS‐CoV‐2 pseudoviruses (wildtype, Alpha, and Beta). Self‐complementary (scAAV5)‐RBD‐plus vaccine elicited strong immune response as well as long‐term humoral immunity, and exhibited NAbs against five typical SARS‐CoV‐2 pseudoviruses (wildtype, Alpha, Beta, Gamma, and Delta) [220]. AAV‐SRBD vaccine, with stabilizing β‐sheet formed in spike protein from Q321 to S591, demonstrates good seroconversion rate, long neutralizing antibody durability, and long‐term protection against SARS‐CoV‐2 [221].

## 7 AAV in Monkeypox Vaccine

Monkeypox virus (Mpox), an enveloped double‐stranded DNA virus of the Orthopoxvirus genus within the Chordopoxvirinae subfamily in the Poxviridae family, could be identified into two clades: Clade I (central African clade with subclades Ia and Ib) and Clade II (west African clade with subclades IIa and IIb) [222, 223]. Mpox causes signs and symptoms, such as rash, fever, sore throat, headache, muscle aches, back pain, low energy, swollen lymph nodes, which usually begin within 1 week and typically last 2–4 weeks [224, 225]. There are three vaccines for Mpox treatment: LC16m8, ACAM2000, and JYNNEOS [226]. LC16m8, a live, attenuated, cell‐cultured smallpox vaccine, was licensed in Japan in the 1970s as a first‐generation smallpox vaccine to prevent human monkeypox, which received immunization recommendation stockpile vaccine from the World Health Organization Strategic Advisory Group in 2013 [227, 228]. ACAM2000, a live‐attenuated, replication‐competent vaccinia virus vaccine, was approved by the US FDA in 2015 as a second‐generation vaccine against smallpox as a post‐exposure prophylactic agent, which was the only available vaccine for Mpox in the U.S.A from 2015 to 2019 [229, 230]. JYNNEOS, also known as IMVAMUNE and IMVANEX, is a Modified Vaccinia Ankara (MVA)‐based live, non‐replicating vaccine, which was approved by the US FDA in 2019 as a two‐dose series, with doses administered 4 weeks apart [231, 232]. WHO has declared Mpox a public health emergency of international concern (PHEIC) twice, the first time in May 2022 and the second time in August 2024 [233]. From Jan 2022 to Aug 2024, over 120 countries have reported mpox with over 100 000 laboratory‐confirmed cases and over 220 deaths among confirmed cases [233].

The CRISPR–Cas system induces double‐strand DNA breaks at specific genomic loci identified by single‐guide RNAs, which has also been studied as an antiviral therapy for HIV [234], hepatitis viruses [235], herpes viruses [236], and human papillomavirus [237]. AAV has proven to be an effective vector for the CRISPR–Cas system for antiviral therapy [238], with the characteristics of broad tissue tropism, safe viral applications, and low integration risk, which provide indication for the success of generating AAV‐based vaccines for Mpox treatment.

## 8 AAV in Clinical Trials

**Figure 9:**
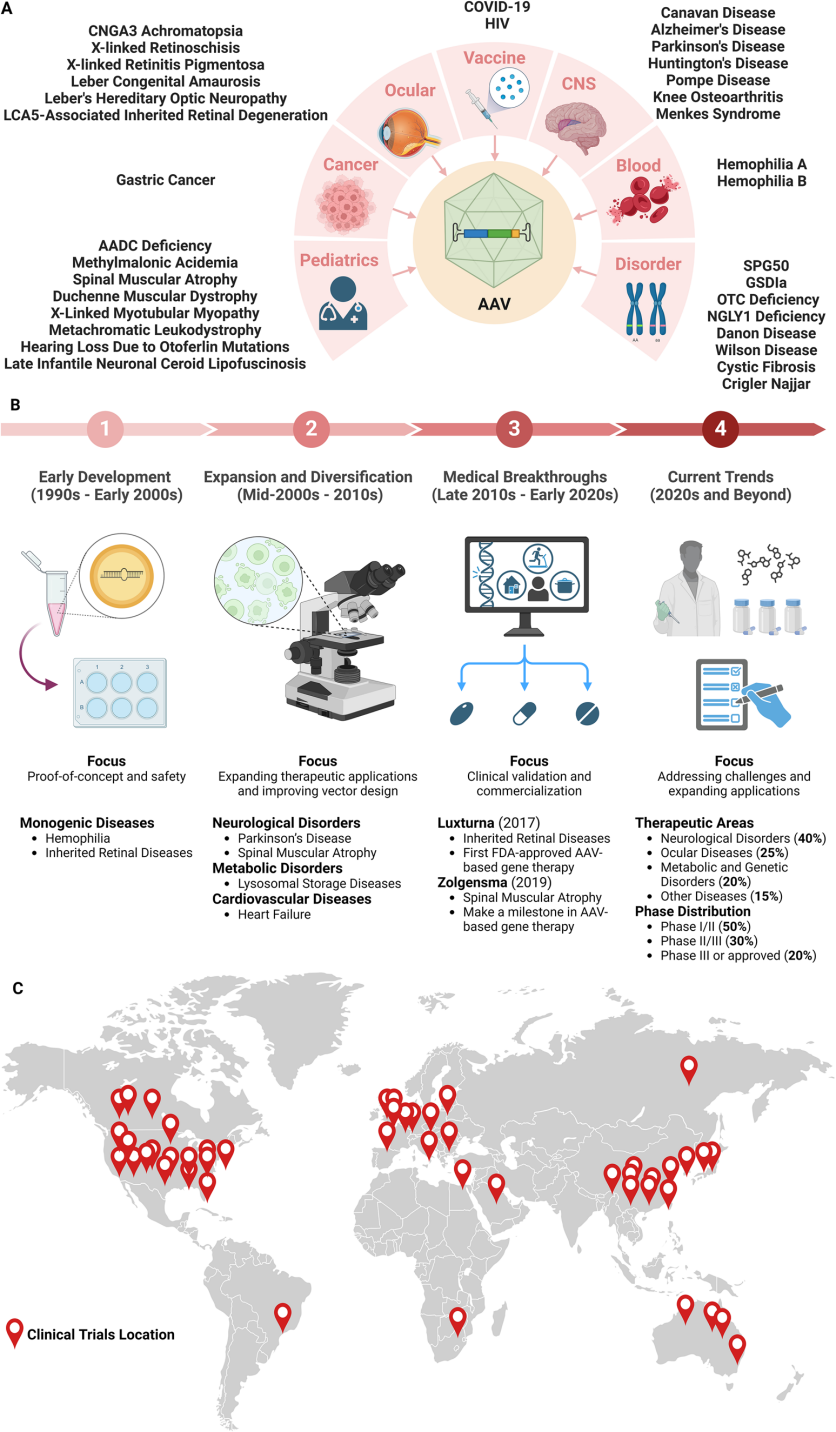
The distributions of AAV clinical trials in different diseases (A), periods (B), and areas (C). The data are summarized from ClinicalTrials.gov. The figure categorizes gene therapy targets into four major groups: ocular diseases (e.g., Achromatopsia, Leber Congenital Amaurosis), cancer and metabolic disorders (e.g., AADC deficiency, Duchenne Muscular Dystrophy), infectious diseases (COVID‐19, HIV) with vaccine development applications, CNS and systemic disorders (e.g., Alzheimer's, Hemophilia, Cystic Fibrosis). The figure outlines the historical progression of AAV gene therapy across four key phases: (1) Early Development (1990s–2000s): Proof‐of‐concept studies focused on monogenic diseases (hemophilia, retinal disorders); (2) Expansion Phase (2000s–2010s): Diversification into neurological, metabolic and cardiovascular applications; (3) Breakthrough Era (2010s–2020s): FDA approvals of Luxturna (2017) and Zolgensma (2019); (4) Current Trends (2020s‐current): Therapeutic area distribution (40% neurological) and clinical phase breakdown (50% Phase I/II trials).

According to ClinicalTrials.gov, there are 146 AAV‐based gene therapy clinical trials, with 130 interventional studies and 16 observational studies. There are different funder/sponsor types for supporting the clinical trials: NIH (14), Industry (107), Other Individuals and Organizations (56). All the clinical trials take gender and age into consideration. For the gender item, 102 clinical trials involve female patients, while 146 clinical trials involve male patients. The patients involved include 64 Children (birth‐17), 129 Adults (18‐64), and 114 Seniors (65+). Out of 146 AAV‐based gene therapy clinical trials recorded, 49 of them were completed, 33 of them were active, 34 of them were recruiting, 6 of them were enrolled by invitation, while 16 were terminated, 4 were not yet recruiting, and the remaining 4 have unknown status or withdrawn. The clinical trials are in different phases: Phase 1 (95), Phase 2 (69), Phase 3 (14), Phase 4 (0), Early Phase 1 or not applicable (13). There are several clinical trials for AAV therapies with completed results (Table 2). We also list some ongoing AAV clinical trials for different diseases in different phases (Table 3). All the clinical trials are distributed in different disease categories (cancer, neurodegenerative, infectious diseases, ocular diseases, blood diseases, pediatric diseases, genetic disorder), different spanning periods (early development, expansion and diversification, breakthroughs and regulatory approvals, current trends), and different geographic regions (USA, Canada, UK, France, Germany, Italy, Spain, Belgium, Bulgaria, Porland, Israel, Saudi Arabia, Australia, China, Japan, South Korea, Brazil, South Africa) (Figure 9).

**Table 2:** Safety and dose‐escalation of AAV in disease clinical trials.

ClinicalTrials.gov ID	AAV vector	Target disease	Phase	Sponsor	Origin country	Study period	Administration
NCT00976352	rAAV1‐CMV‐GAA	Pompe Disease	I/II	University of Florida	USA	July 2009 to September 2018	Intramuscular injection at low dose (1 × 10^12^ vg/mL) and high dose (5 × 10^12^ vg/mL).
NCT00749957	rAAV2‐CB‐hRPE65	Leber Congenital Amaurosis	I/II	Applied Genetic Technologies Corp	USA	September 2008 to October 2017	Unilateral subretinal injection at low dose (1.8 × 10^11^ vg/mL) and high dose (6 × 10^11^ vg/mL).
NCT02781480	AAV2/5‐OPTIRPE65	Leber Congenital Amaurosis	I/II	MeiraGTx UK II Ltd	UK	April 2016 to June 2021	Unilateral subretinal injection at low dose (1 × 10^11^ vg/mL), intermediate dose (3 × 10^11^ vg/mL), high dose (1 × 10^12^ vg/mL).
NCT02416622	rAAV2tYF‐CB‐hRS1	X‐linked Retinoschisis	I/II	Applied Genetic Technologies Corp	USA	April 2015 to June 2023	Intravitreal injection at low dose (1 × 10^11^ vg/eye), intermediate dose (3 × 10^11^ vg/eye), high dose (6 × 10^11^ vg/eye).
NCT00430768	rAAV1‐CB‐hAAT	Alpha‐1 Antitrypsin Deficiency	I	University of Massachusetts	USA	January 2007 to December 2016	Intramuscular injection at low dose (6.9 × 10^12^ vg/kg), intermediate dose (2.2 × 10^13^ vg/kg), high dose (6 × 10^13^ vg/kg).
NCT01054339	rAAV1‐CB‐hAAT	Alpha‐1 Antitrypsin Deficiency	II	Applied Genetic Technologies Corp	USA	January 2010 to July 2016	Intramuscular injection at low dose (6 × 10^11^ vg/kg), intermediate dose (1.9 × 10^12^ vg/kg), high dose (6 × 10^12^ vg/kg).
NCT02168686	AAVrh.10hA1AT	Alpha‐1 Antitrypsin Deficiency	I/II	Adverum Biotechnologies Inc.	USA	June 2014 to September 2023	Intravenous injection at low dose (8 × 10^13^ vg/kg), intermediate dose (4 × 10^14^ vg/kg), high dose (1.2 × 10^15^ vg/kg).
NCT02341807	AAV2‐hCHM	Choroideremia	I/II	Spark Therapeutics Inc.	USA	January 2015 to January 2024	Unilateral subretinal injection at low dose (5 × 10^10^ vg/eye) and high dose (1 × 10^11^ vg/eye).
NCT00985517	AAV2‐Neurturin	Parkinson's Disease	I/II	Sangamo Therapeutics	USA	September 2009 to April 2020	Direct neurosurgeon injection at low dose (9.4 × 10^11^ vg/mL) and high dose (2.4 × 10^12^ vg/mL).
NCT00400634	AAV2‐Neurturin	Parkinson's Disease	II	Sangamo Therapeutics	USA	November 2016 to November 2022	Bilateral intraputaminal administration at dose of 5.4 × 10^11^ vg/mL.
NCT02991144	scAAV8OTC	Late‐Onset Ornithine Transcarbamylase Deficiency	I/II	Ultragenyx Pharmaceutical Inc	USA	December 2016 to January 2023	Peripheral intravenous infusion at low dose (2 × 10^12^ GC/kg), intermediate dose (6 × 10^12^ GC/kg), high dose (1 × 10^13^ GC/kg).
NCT03517085	AAV8G6PC	Glycogen Storage Disease Type Ia	I/II	Ultragenyx Pharmaceutical Inc	USA	April 2018 to October 2022	Peripheral intravenous infusion at low dose (2 × 10^12^ GC/kg), high dose (6 × 10^12^ GC/kg).
NCT01976091	scAAVrh74.tMCK.hSGCA	Limb‐Girdle Muscular Dystrophy, Type 2D	I/II	Sarepta Therapeutics Inc.	USA	July 2013 to April 2023	Isolated Limb Infusion at low dose (1 × 10^12^ vg/kg), intermediate dose (2 × 10^12^ vg/kg), high dose (6 × 10^12^ vg/kg).

**Table 3:** Ongoing AAV clinical trials for different diseases.

ClinicalTrials.gov ID	Drug	Target disease	Phase	Start date	End date	Admistration
NCT03370172	BAX 888	Hemophilia A	I/II	2017‐11	2025‐04	Single intravenous infusion
NCT03588299	DTX201	Hemophilia A	I/II	2018‐07	2026‐11	Single intravenous administration
NCT04684940	BMN 270	Hemophilia A	I/II	2020‐11	2029‐04	Single dose intravenous injection
NCT04135300	BBM‐H901	Hemophilia B	I	2019‐10	2039‐12	Single dose intravenous injection
NCT01687608	AskBio009	Hemophilia B	I/II	2012‐08	2030‐01	Single dose intravenous injection
NCT02496273	CTL	Stage IV gastric cancer	I	2015‐07	2030‐12	Patients receive CEA‐specific CTLs
NCT03882437	RP‐A501	Danon disease	I	2019‐03	2025‐03	Single intravenous infusion
NCT06092034	RP‐A501	Danon disease	II	2023‐10	2029‐09	Single intravenous infusion
NCT02935517	AGTC‐402	CNGA3 achromatopsia	I/II	2016‐10	2026‐08	Subretinal injection
NCT04783181	BBP‐631	Congenital adrenal hyperplasia	I/II	2021‐03	2029‐02	Single dose intravenous injection
NCT05835895	GNSC‐001	Knee osteoarthritis	I	2023‐04	2029‐05	Single intra‐articular injection
NCT05541627	AB‐1001	Huntington's disease	I/II	2022‐09	2029‐12	Single intracerebral bilateral injection

## 9 Adverse Effects and Mitigation Strategies

AAV‐based gene therapy has demonstrated significant potential in addressing various genetic disorders. Nonetheless, it is accompanied by possible adverse effects, including immune reactions, off‐target impacts, and thrombotic microangiopathy. The primary adverse effects and corresponding mitigation strategies related to AAV‐based gene therapy are outlined below.

### 9.1 Immune Response

The immune reaction to AAV‐based gene therapy is a complex and multifaceted issue, which can be categorized into innate and adaptive immune responses (humoral and cellular immunity). The adverse effects associated with immune responses include pre‐existing immunity, capsid‐specific immune reactions, transgene‐specific immune responses, innate immune activation, liver toxicity, and complement activation. Many individuals possess pre‐existing NAbs due to prior natural exposure to AAV, which can hinder vector transduction and diminish therapeutic efficacy [239]. Strategies to address pre‐existing immunity include screening for NAbs, using empty capsid decoys, serotype switching, and plasmapheresis [240]. The AAV capsid can trigger both antibody‐mediated (humoral) and cell‐mediated (cellular) immune responses, which can be mitigated through capsid engineering to evade immune detection and by coating the capsid with polymers, peptides, or proteins to reduce immune recognition [241, 242]. Transgene‐specific immune responses, which target the protein product encoded by the AAV‐delivered transgene, can be minimized using codon‐optimized sequences, tissue‐specific promoters, and immune‐modulatory molecules [243, 244]. AAV vectors can also activate innate immune pathways, such as Toll‐like receptor (TLR) signaling, leading to inflammation and cytokine release [245]. This can be countered by administering TLR pathway inhibitors and using highly purified AAV preparations [246]. Liver toxicity, often indicated by elevated liver enzymes, can be managed through liver‐specific immunosuppressive strategies and regular monitoring of liver enzymes and bilirubin [247, 248]. Complement activation, which results in the release of pro‐inflammatory cytokines and chemokines, can be temporarily suppressed using pharmacological inhibitors like the C5 inhibitor eculizumab during AAV administration [249, 250].

### 9.2 Off‐Target Effects

Off‐target effects refer to unintended gene modifications or interactions occurring outside the intended target site, posing significant challenges in AAV‐based gene therapy. These effects include oncogenesis, inflammation, immune‐mediated tissue damage, toxicity or dysfunction in non‐target tissues, silencing or activation of non‐target genes, and loss of therapeutic efficacy [251, 252, 253, 254]. Self‐complementary AAV (scAAV) can achieve faster transgene expression, potentially reducing the required dose and minimizing off‐target effects [255]. Optimizing AAV delivery methods, such as intravenous, intramuscular, intraocular, intrathecal, intranasal, and local administration, can enhance tissue targeting and reduce off‐target expression [256]. AI/ML computational tools can predict potential off‐target sites and refine vector design, which can then be validated in cell cultures and animal models [257]. Developing biomarkers can aid in early detection of off‐target activity, allowing for timely adjustments to treatment strategies [258]. For AAV‐delivered CRISPR/Cas9 components, the risk of off‐target DNA cleavage can be reduced by using high‐fidelity Cas variants, base editing, and prime editing [259]. Long‐term patient monitoring, follow‐up, and risk‐benefit analysis are essential to ensure the safety and efficacy of AAV‐based gene therapies [260].

### 9.3 Thrombotic Microangiopathy (TMA)

TMA is a rare but severe complication associated with AAV‐based gene therapies, characterized by thrombocytopenia, microvascular thrombosis, hemolytic anemia, and end‐organ damage, particularly affecting the brain and kidneys [261, 262, 263]. TMA following AAV gene therapy is linked to immune activation, complement dysregulation, and endothelial injury triggered by the vector or transgene product. This can lead to a range of clinical and laboratory presentations, including hemolytic anemia (decreased hemoglobin, elevated lactate dehydrogenase, and increased bilirubin), thrombocytopenia (reduced platelet count), renal dysfunction (elevated serum creatinine, proteinuria, and hematuria), acute kidney injury (elevated creatinine, proteinuria, hematuria), and neurological symptoms (confusion, headaches, seizures, and focal neurological deficits) [264, 265]. Early recognition and intervention are crucial for improving patient outcomes, with monitoring of platelet counts, lactate dehydrogenase, haptoglobin, and renal function being essential [266]. Preventive measures to reduce the risk of TMA in patients receiving AAV gene therapy include assessing pre‐existing immunity, optimizing vector doses, providing supportive care, inhibiting complement activation, performing plasma exchange, administering immunosuppressive therapy, and conducting post‐treatment monitoring [267, 268].

## 10 Future Direction

**Figure 10:**
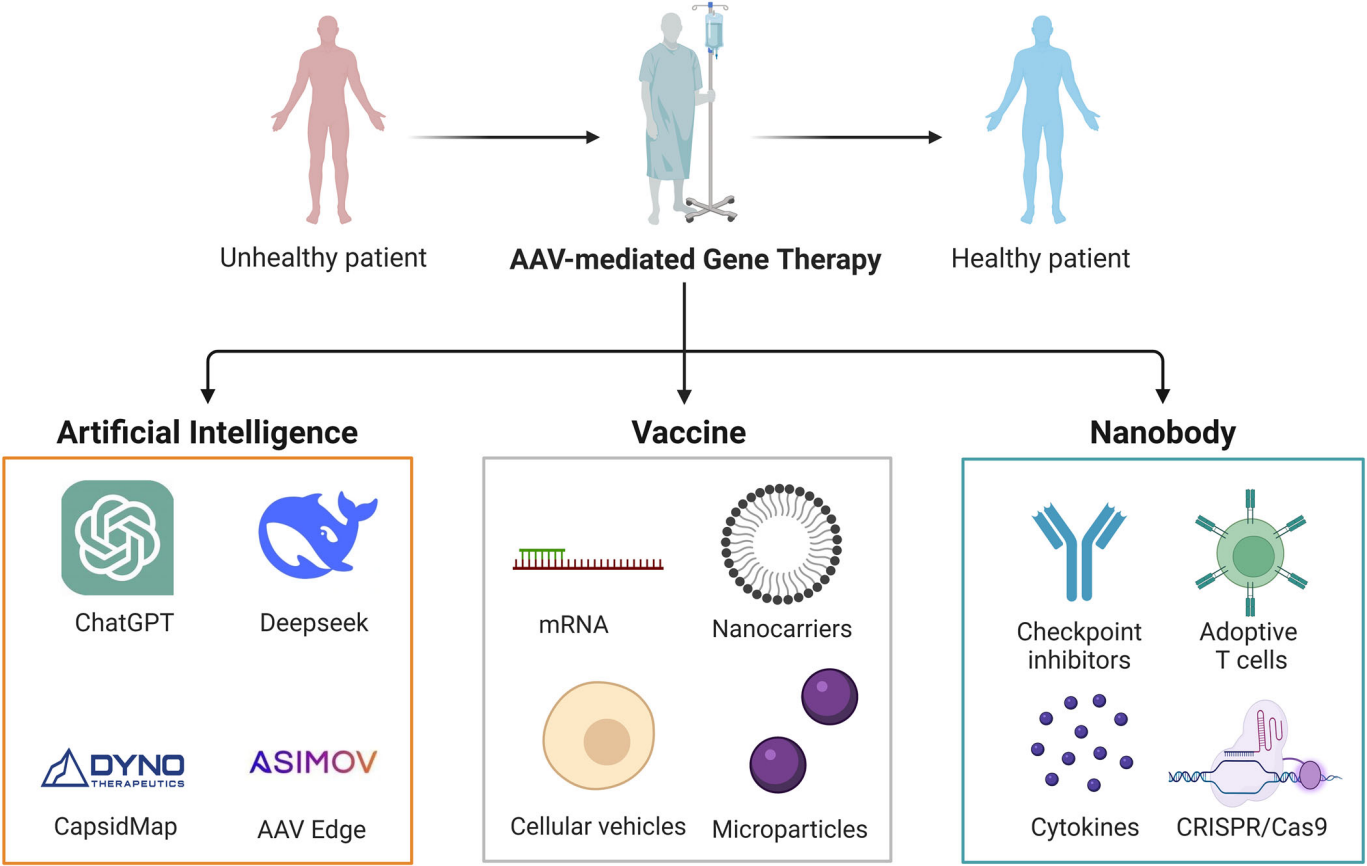
The Future direction of AAV‐mediated gene therapy will be accelerated and boosted by the application of artificial intelligence, vaccines, and nanobodies. The figure highlights three cutting‐edge therapeutic platforms: (1) Artificial Intelligence applications (ChatGPT, Deepseek) for AAV capsid optimization (CapsidMap, AAV Edge); (2) Vaccine technologies including mRNA platforms and novel delivery systems (nanocarriers, cellular vehicles); (3) Nanobody‐based therapies combined with checkpoint inhibitors, adoptive T cells, and CRISPR/Cas9 gene editing.

A deeper understanding of AAV basic biology and host‐virus interaction can lead to druggable target discovery. The continuous development of AI, vaccines, and nanobodies will provide an arsenal for combating and controlling emerging diseases. The combination of prevention treatment with clinical trials on drug discovery in a timely fashion will be extremely important (Figure 10).

### 10.1 Artificial Intelligence

In 2023, the release of Chat Generative Pre‐trained Transformer (ChatGPT), a large language model (LLM) built by OpenAI, generated text based on written prompts, which triggered an AI boom in large tech companies such as Meta, Google, and X [269, 270]. ChatGPT features multiple layers of self‐attention and feed‐forward neural networks as well as accelerates the applications in medicine, such as identifying potential research topics, updating novel research developments, assessing medical education outcomes, providing clinical consultation/decision support, and managing patient health records, which could bypass traditional plagiarism detection methods and possess novel treatment options [271, 272, 273, 274, 275]. In 2024, the release of Sora, a state‐of‐the‐art AI model developed by OpenAI, generates lifelike and imaginative visual scenes based on text prompts, which rests on LLM and artificial video generation techniques [276, 277]. Sora includes several procedures, such as processing text content, understanding text meaning and context, translating text into images, and forming a video sequence, which could enhance clinician education, simulate surgical training, and facilitate remote consultation [278]. In 2025, the release of DeepSeek‐R1, a partially open‐source reasoning model designed by DeepSeek AI, was evaluated by data‐driven scientific tasks [279, 280]. DeepSeek‐R1 offers free access, enables offline use, and enhances data security by data interpretation, manuscript drafting, open‐weight customization, as well as trade‐offs between innovation and practicality [281, 282].

However, all the AI tools have technical, legal, and ethical risks, such as inaccurate responses, repeated phrases, fabricating images, scientific misconduct, copyright laws infringement, medico‐legal complications, which hinder their applications in medicine, especially AAV‐mediated gene therapy and drug discovery [283, 284]. To identify and manage AI risks, the U.S. Department of State, as well as the National Institute of Standards and Technology (NIST), released related profiles and documents. The U.S. Department of State released “Risk Management Profile for Artificial Intelligence and Human Rights” on July 25, 2024, which serves as a practical guide for organizations to design, develop, deploy, use, and govern AI in a manner consistent with respect for international human rights [285]. NIST released “Artificial Intelligence Risk Management Framework: Generative Artificial Intelligence Profile” on July 26, 2024, which helps organizations identify and manage unique AI risks for aligning with best research and clinical goals and priorities [286].

### 10.2 Vaccine

Vaccination has played a crucial role in preventing diseases, with early development in Asia and late development in Europe [287, 288]. Vaccines could help to people of all ages live longer and healthier by preventing more than 20 life‐threatening diseases, which includes Cervical Cancer, Cholera, COVID‐19, Diphtheria, Hepatitis B, Influenza, Japanese Encephalitis, Malaria, Measles, Meningitis, Mumps, Pertussis, Pneumonia, Polio, Rabies, Rotavirus, Rubella, Tetanus, Typhoid, Varicella, and Yellow Fever [289]. The WHO estimates that currently available vaccines prevent 3.5–5 million deaths worldwide annually from diseases such as Diphtheria, Influenza, Measles, Tetanus, and Pertussis [289]. During all the vaccines, inactivated vaccines are the quickest option for vaccine development, which are combined with multiple chemical and physical methods, such as β‐propiolactone, formalin, formaldehyde, and UV [290].

Despite vaccine successes in preventing various diseases, there are still considerable hurdles to overcome in vaccine development. The efficacy and safety of vaccines are affected by multiple variables, including high virus variability, existing virus immunity, and underlying health circumstances [291]. The immune response to vaccines is influenced by several factors, such as sex, age, lifestyle, genetic variations, and medical conditions [291]. The stepwise vaccine development in and timely commercial availability could be achieved by the following procedures: (1) Documenting the observations of protecting individuals and general population against infectious diseases; (2) Performing childhood and adult mass‐vaccination programs to confer herd immunity and eradicate specific infections; (3) Exploring single‐cycle and replication‐deficient viral vectors have been explored as alternative options to offer high safety profile and elicit low immune response; (4) Conducting targeted international collaboration in the areas of political will, worldwide leadership and role models to promote vaccine acceptance, resolve vaccine hesitancy and accelerate vaccine development [292, 293, 294].

### 10.3 Nanobody

Nanobodies (Nbs), with small size, high specificity, excellent solubility, superior stability, and strong tissue penetration, are composed of four framework regions (FR1–4) alternated with three complementarity determining regions (CDR1–3), which could be easily produced in an appropriate expression system such as *E. coli*, *S. cerevisiae*, *P. pastoris* [295, 296]. Nbs gain increasing acceptance as diagnostic proteins and therapeutical tools in immuno‐oncology due to their unique functional and structural properties. Several anti‐PD‐1/PD‐L1/CTLA‐4 Nbs with high specificity and affinity have been screened and generated, such as Nb‐Fc. ^68^Ga‐labeled NOTA‐mal‐hPD‐L1 Nb, and Nb36, which could be a promising candidate for cancer immunotherapy without undesirable cytotoxicity [297, 298, 299]. Nanobody‐based CAR‐T cells can function as an antitumor agent in tumor microenvironment and tumor xenograft models, such as CD19 CAR‐T, CD70 CAR‐T, CD72 CAR‐T, CD105 CAR‐T, which are engineered by CRISPR/Cas9 and in silico techniques [300, 301, 302, 303]. The tumor‐specific DC/tumor‐fusion cell (FC)/mRNA vaccine in combination anti‐PD‐1/PD‐L1/CTLA‐4 Nbs (PD‐1 Nb20, ^99m^Tc‐Nbs C3, Nb36) could improve the activation, proliferation, cytokine secretion, and tumor cell cytotoxicity of CD8^+^ T‐cells [304, 305, 306].

The combination of AAV‐mediated gene therapy with AI/Vaccine/Nanobody has been a viable therapeutic strategy in the biomedical field, which can be used to manipulate and deliver genetic targets and materials in a tissue‐specific manner. Dyno Therapeutics released the CapsidMap Platform in May 2020, which utilized AI to systematically and rapidly optimize AAV capsids to improve targeting ability, payload size, immune evasion, and manufacturability [307]. As a breakthrough CNS‐targeted AAV gene delivery vector with best‐in‐class potential, Dyno bCap 1 Capsid was launched in May 2023, which was created by CapsidMap Platform to achieve high transduction efficiency, on‐target specificity, and yield production [308]. In September 2024, Asimov announced the launch of the AAV Edge System, the first comprehensive platform to enable end‐to‐end optimization, which provides a single access point to best‐in‐class tools [309]. The capsid‐engineered AAV vector‐based vaccine, with genetic insertion of major histocompatibility complex Class I/II (MHC Class I/II), could induce robust and long‐lasting antigen‐specific humoral and CD4^+^ and CD8^+^ T cell immune responses after single intramuscular administration, serving as a novel vaccine platform for cancer immunotherapy [310]. Ferring Pharmaceuticals’ nadofaragene firadenovec (Adstiladrin), the first AAV vector‐based gene therapy for the treatment of bladder cancer, was approved by the FDA in December 2022 [311]. In September 2024, FDA cleared an IND application for the first‐ever clinical trial of Vironexis Biotherapeutics’ AAV‐delivered cancer immunotherapy VNX‐101 [312]. Nbs are single immunoglobulin variable domains from camelids heavy chain antibodies, which could be inserted into AAV2 VP1 capsid and incorporated into the capsids of AAV1, AAV8, and AAV9 [313]. AAV‐mediated expression of nanobody is applied in preclinical studies of different diseases with different serotypes, such as cancer (AAV1 and AAV8), cardiovascular diseases (AAV9), genetic diseases (AAV8 and AAV9), infectious diseases (AAV8 and AAV9), and neurodegenerative diseases (AAV5) [314].

However, there still needs to be preclinical and systematic efficacy and safety evaluation for AAV‐mediated gene therapy both in mice and NHP to provide theoretical basis for achieving clinical translation and fulfilling drug discovery, which included neural degeneration, vector biodistribution, potential toxicity, expression profile, and immune response in on‐ and off‐target tissues. There are several items involved in maximizing AAV's efficiency and safety, such as vector serotype choice, clinical phase measurements, and long‐term follow‐up. AAV‐1, 5, and 7 could transduce murine skeletal muscle much more efficiently than AAV‐2, with significant expression increase ranging from 2‐ to 1000‐fold [315]. As promising vectors for the lysosomal enzyme arylsulfatase A (ARSA) delivery, AAVs can trans‐synaptically transduce neurons through anterograde transport by AAV‐1, 2, 5, 6 rather than AAV‐8, 9 [316, 317]. AAV‐LK03 exhibits a 10‐fold transduction efficacy increase in human hepatocytes compared to AAV8 in Fah^−/−^/Rag2^−/−^/Il2rg^−/−^(FRG) mice [318]. Vector safety could be evaluated before AAV administration and at designated follow‐up visits after administration by conducting general physical examination as well as classical clinical and laboratory tests. For patients with Leber's Congenital Amaurosis, at least four measurements should be taken 1 month post‐injection of AAV vectors, which include pupillary light reflex evaluation, visual acuity testing, Goldmann visual‐field examination, retinal degeneration, and nystagmus testing [319]. Meanwhile, long‐term efficiency and safety follow‐up studies are essential for AAV applications in histopathological, biochemical, and clinical areas. AAV‐mediated retinal gene therapy in patients with choroideremia showed therapeutic effects at the 6‐month and 3.5‐year follow‐up treatment [320, 321].

Consequently, future research should focus on creating AI‐driven AAV capsid engineering tools and packaging capacity/immune response prediction methods evolving from current basic programming to support the learning of more advanced concepts. Meanwhile, researchers and industry professionals should collaborate to develop and standardize AAV‐based passive immunization with nanobody as a cost‐effective and widely adoptable vaccine platform in terms of site mutation, packaging efficiency, biodistribution potential, immune response, and disease models.

## Author Contributions

T.J. provided funding, conceived the topic, and edited the manuscript. L.Y. conceived the topic, designed the main idea, drafted the original manuscript, and the figures. All authors reviewed and approved the final version of the manuscript.

## Data Availability

Data sharing is not applicable to this article as no new data were created or analyzed in this study.
